# Gut microbiota-derived metabolites and host interactions in fibrotic diseases: mechanisms, cross-organ signatures, and therapeutic opportunities

**DOI:** 10.3389/fmicb.2026.1893651

**Published:** 2026-09-01

**Authors:** Yang Pan, Chang Shu, Yuqing Mei, Yan Hu, Yueping Qiu, Luo Fang

**Affiliations:** 1 Postgraduate Training Base Alliance of Wenzhou Medical University (Zhejiang Cancer Hospital), Hangzhou, Zhejiang, China; 2 Zhejiang Cancer Hospital, Hangzhou Institute of Medicine (HIM), Chinese Academy of Sciences, Hangzhou, Zhejiang, China

**Keywords:** gut microbiota, gut–organ axis, microbial metabolites, multiorgan fibrosis, organ-specific regulation, short-chain fatty acids

## Abstract

Fibrosis is a common end-stage pathological change across multiple chronic organ injuries and a primary driver of global organ failure and high mortality. Accumulating evidence indicates that the gut microbiota and its metabolites can bidirectionally regulate fibrosis progression through the gut–organ axis; however, a unified and systematic theoretical framework remains lacking. This review synthesizes evidence from six major organs—the liver, biliary tract, intestine, lung, heart, and skin—and proposes a “shared core pathway–organ-specific differentiation” dual-layer theoretical framework for microbiota-mediated multiorgan fibrosis. In this shared layer, short-chain fatty acid signaling, the tryptophan–AhR pathway, intestinal barrier disruption with microbial translocation, and bile acid metabolic disorders constitute the common metabolic–immune basis across organs. Animal intervention studies, including germ-free colonization, metabolite reconstitution, and gene knockout experiments, have established the causal regulatory effects of these four pathways, whereas human cross-sectional and cohort studies can reveal only statistical associations between metabolites and fibrosis stage without establishing causality. At the specific layer, organs differ in their anatomical exposure patterns, resident cell types, and local microenvironments, leading to divergent responses to identical microbial signals and the formation of organ-specific signature pathways: the hepatobiliary system features the bile acid/FXR axis as its core regulatory mechanism; the heart possesses a unique TMAO–JAK2–STAT3 profibrotic axis; the lung utilizes palmitoylethanolamide as a specific protective mediator; and the skin integrates circulating metabolic signals with Piezo1 mechanosensation to form a dual regulatory network. This dual-layer framework provides a unified explanation for the divergent effects of identical metabolites across organs, offering both theoretical support for broad-spectrum microbiota-based antifibrotic interventions and new perspectives for precision stratified diagnosis and treatment of fibrosis in individual organs. Future studies should move beyond observational research by leveraging large-scale prospective cohorts and standardized randomized controlled trials to complete causal validation, thereby translating microbiota-targeted strategies from animal-based mechanistic research into clinical practice for the precision prevention and treatment of multiorgan fibrosis.

## Graphical Abstract

**Figure:**
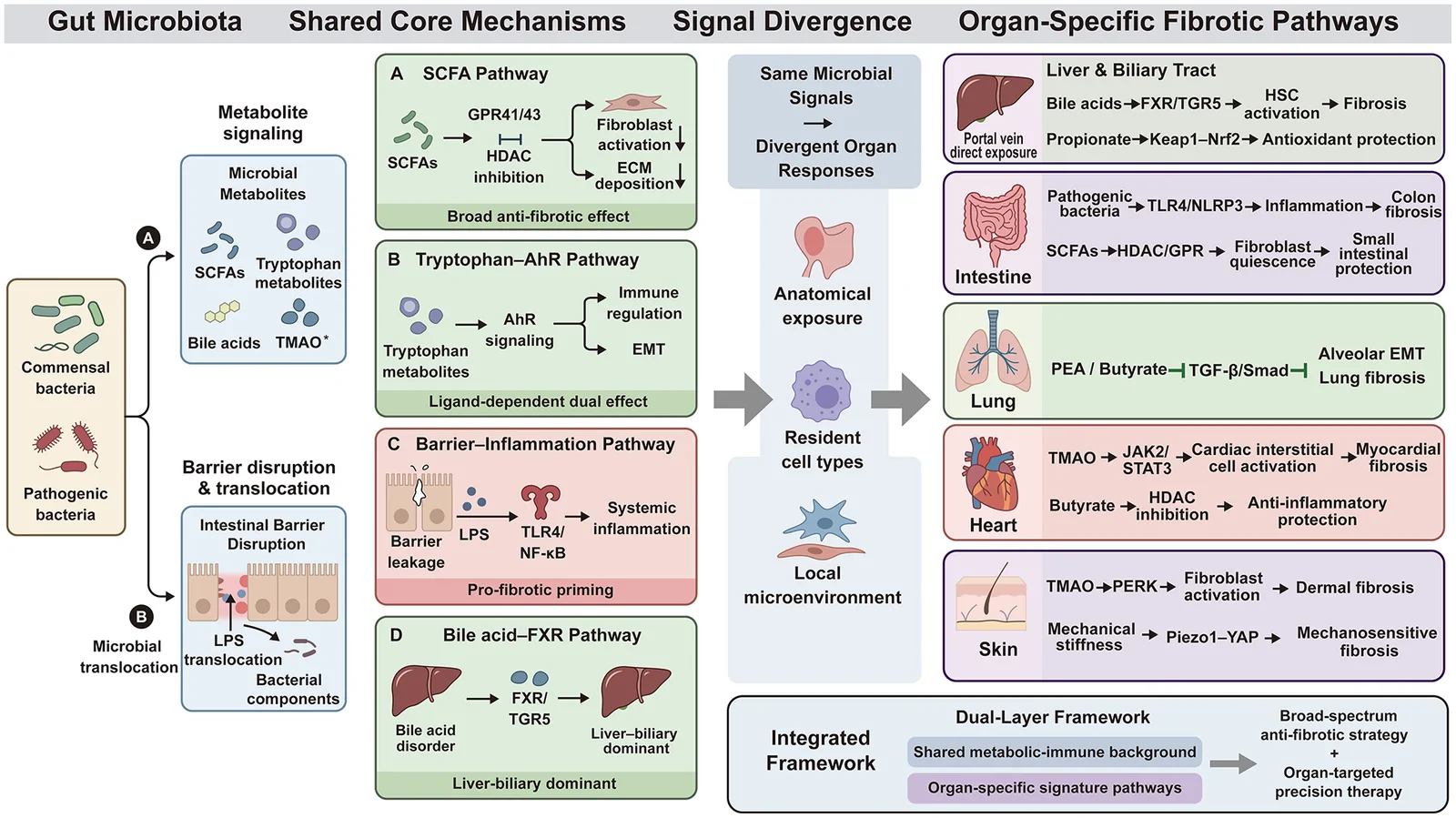
This schematic proposes a dual-layer framework describing gut microbiota-driven multi-organ fibrosis. Commensal and pathogenic bacteria transmit signals via two routes: metabolite signaling and intestinal barrier disruption accompanied by microbial translocation. Four shared core mechanisms (SCFA pathway, tryptophan–AhR pathway, barrier–inflammation pathway, and bile acid–FXR pathway) mediate gut-derived signaling. Identical microbial metabolites trigger divergent fibrotic outcomes across organs, mainly determined by anatomical exposure, resident cell populations, and local tissue microenvironment. *TMAO acts as a fibrotic mediator independent of the four shared pathways. This dual-layer paradigm, comprising shared metabolic-immune background and organ-specific signature pathways, inspires therapeutic strategies combining broad-spectrum anti-fibrotic intervention and organ-targeted precision therapy.

## 1 Introduction

Fibrosis is a shared hallmark of the terminal stages of chronic inflammation, metabolic disorders, tissue injury, and other pathological processes and constitutes a leading cause of organ failure worldwide (Wynn and Ramalingam, 2012; Rockey et al., 2015; Henderson et al., 2020). It can extensively involve multiple vital organs, including the liver, biliary tract, intestine, lungs, heart, and skin. The core pathological changes include aberrant activation of fibroblasts, their transformation into myofibroblasts, and excessive deposition and abnormal cross-linking of extracellular matrix (ECM) components such as collagen and fibronectin, ultimately resulting in the structural degradation and functional failure of the affected organs (Wynn and Ramalingam, 2012; Rockey et al., 2015). Statistics indicate that approximately 45% of deaths from chronic diseases worldwide are directly attributable to fibrosis (Rockey et al., 2015): The 5-year survival rate for patients with liver fibrosis progressing to cirrhosis is less than 30%; the median survival of idiopathic pulmonary fibrosis patients is only 2–3 years (Raghu et al., 2015); and complications related to intestinal and cardiac fibrosis also significantly increase mortality risk. Although some progress has been made in elucidating the pathological mechanisms of single-organ fibrosis in recent years, effective targeted therapies that can reverse or slow fibrotic progression in clinical practice remain lacking; moreover, the systemic regulatory mechanisms underlying multiorgan fibrosis have not been fully clarified, posing major challenges for clinical diagnosis and treatment.

The human gut harbors approximately 10^14^ microorganisms, whose collective functional genes outnumber the human genome by approximately 150-fold (Qin et al., 2010; Bäckhed et al., 2005). The gut microbiota participates in host nutritional metabolism, immune homeostasis, maintenance of the intestinal epithelial barrier, and other physiological processes, thereby participating extensively in regulating systemic homeostasis (Tang et al., 2017). Cross-sectional population cohort studies have shown that compositional dysbiosis (abnormal abundance of dominant taxa and reduced microbial diversity) and metabolic imbalance (decreased short-chain fatty acids, accumulation of trimethylamine N-oxide, and dysregulation of the bile acid pool) are statistically associated with the severity of fibrosis in patients with various chronic diseases (Nogal et al., 2021; Shi et al., 2023; Loomba et al., 2017). Animal intervention experiments have further shown that these microbial and metabolomic changes can directly promote or inhibit fibrotic progression through specific molecular pathways, providing experimental support for causal regulation (Zhang et al., 2025; Zhao et al., 2023; Wang et al., 2022).

With deepening research on the “gut–organ axis” regulatory network, the systemic regulatory role of the gut microbiota has been further elucidated: The microbiota links intestinal microecological status to systemic organ function via trans-organ pathways such as the gut–liver axis, gut–lung axis, gut–skin axis, and gut–heart axis through metabolite-mediated signaling, cytokine signals, and immune cell trafficking (Lin et al., 2025; Tripathi et al., 2018; Dang and Marsland, 2019; O'Neill et al., 2016). This regulatory mechanism is particularly critical in the progression of multiorgan fibrosis. To date, animal experiments employing germ-free mouse colonization, fecal microbiota transplantation and specific strain interventions have provided experimental evidence for the causal role of the gut microbiota and its metabolites in fibrosis of the liver, heart, lung, skin and other organs (Zhang et al., 2025; Oguri et al., 2024; Park et al., 2024; Kim et al., 2022; Zhou et al., 2025); cross-sectional population cohorts and case–control studies have further revealed statistical correlations between microbial composition/metabolomic profiles and fibrosis severity (Loomba et al., 2017; Stec et al., 2023; Chang et al., 2025).

The regulatory network of the gut microbiota operates throughout the entire process of fibrogenesis. Animal studies indicate that dysbiosis compromises intestinal barrier integrity, inducing a “leaky gut,” through which intestinal endotoxins and bacterial toxins enter the circulation, activate Toll-like receptor (TLR) signaling, and initiate a systemic chronic inflammatory cascade, which in turn induces aberrant fibroblast activation in multiple organs (Saha et al., 2025; Oami et al., 2025). Beneficial microbial metabolites such as short-chain fatty acids (SCFAs) can bidirectionally modulate inflammation and fibroblast function by activating G protein-coupled receptors or by inhibiting histone deacetylase activity, exerting broad-spectrum anti-inflammatory and antifibrotic effects in various animal models (Trompette et al., 2014; Inamoto et al., 2023; Stilling et al., 2016). On the human side, cross-sectional and prospective cohort observations have repeatedly verified statistical associations between specific taxa/metabolites and fibrosis severity, but their causal relationships and clinical interventional value remain to be confirmed by interventional trials (Loomba et al., 2017; Stec et al., 2023; Chang et al., 2025).

Notably, no single microbial strain that simultaneously regulates fibrosis in all organs has yet been identified; however, owing to differences in anatomical structure and physiological microenvironments among organs, the fibrotic processes in different organs exhibit both shared regulatory patterns mediated by the gut microbiota and organ-specific regulatory features at the levels of microbial composition and signaling pathways (Lin et al., 2025; Budden et al., 2017). Nevertheless, the literature has not systematically articulated this “shared–specific” regulatory network.

Compared with existing reviews, this article has three novel aspects. First, most current fibrosis reviews focus on a single organ or a single metabolite pathway, lacking an integrative framework that reconciles multiorgan common mechanisms with organ-specific regulation. Second, many reviews summarize signaling pathways only from a mechanistic perspective without systematically comparing the differential organ distributions of the metabolites themselves, making it difficult to explain the divergent effects of the same metabolite across different organs. Third, the evidence levels for common mechanisms and organ-specific features are often conflated, obscuring the evidence gap between “causal discovery in animals” and “observational associations in humans.” To address these deficiencies, we construct a dual-layer “commonality–specificity” theoretical framework: at the common level, we integrate four major trans-organ metabolic–immune pathways; at the specific level, we identify organ-unique target cells and mediator molecules. We also introduce a two-dimensional horizontal comparison of metabolite weights and pathway weights, distinguishing broad-spectrum effects from organ-restricted effects of the same metabolite. The core goal of this framework is to unify the otherwise fragmented microbiota–fibrosis evidence scattered across organ-specific subfields into a comparable analytical system so that the broad-spectrum intervention potential of common mechanisms and the precision-targeting value of organ-specific mediators are systematically presented along the same evidence chain, thereby providing a clear theoretical roadmap for future translational research from animal causal discovery to human clinical validation.

Through a systematic synthesis of the evidence on microbiota regulation in multiorgan fibrosis, we propose a central academic viewpoint: the regulation of multiorgan fibrosis by the gut microbiota follows a dual-layer “commonality–specificity” framework—the four major shared mechanisms (SCFAs, AhR, intestinal barrier, and bile acids/FXR) constitute a common “metabolic–immune background” for cross-organ fibrotic susceptibility, whereas owing to differences in anatomical exposure patterns, cellular composition, and local microenvironments, each organ produces directional deviations in response to the same shared signals (Wynn and Ramalingam, 2012; Henderson et al., 2020; Lin et al., 2025). On the basis of this framework, we further introduce the concept of “organ-specific mediators,” i.e., unique signaling molecules that have been verified by animal intervention experiments to play a core regulatory role only in a specific organ [e.g., palmitoylethanolamide [PEA] in the lung (Zhou et al., 2025), the Piezo1 mechanosensitive channel in the skin (Liao et al., 2025), and the TMAO-JAK2-STAT3 axis in the heart (Yang X. et al., 2025)]. Statistical correlations between these molecules and fibrosis complications in the corresponding organs have been observed in current population cohorts, but human interventional trials to validate their clinical pathogenic or protective effects remain lacking; however, their causal potential as organ-specific regulatory pathways has been preliminarily validated in animal models. The clinical importance of this framework is that panfibrotic intervention strategies should prioritize targeting common mechanisms, whereas organ-specific interventions require identification of the dominant pathways and unique mediators in each organ.

On this basis, this review integrates recent advances in the fields of multiorgan fibrosis and gut microecology, systematically elucidates the shared mechanisms and core microbial taxa by which the gut microbiota and its metabolites regulate multiorgan fibrosis and then examines the organ-specific regulatory features and pathways in fibrosis of each organ. At the common level, antifibrotic taxa (such as Akkermansia muciniphila, Lactobacillus, Bifidobacterium, Lachnospiraceae, Bacteroides, and Ruminococcaceae) and profibrotic taxa (such as Enterobacteriaceae, Proteus, Staphylococcus aureus, and adherent-invasive Escherichia coli) exert consistent protective or detrimental effects across multiple organs—including the liver, lung, intestine, and skin—through four core pathways: metabolite-mediated signaling, inflammatory regulation, immune modulation, and maintenance of intestinal barrier homeostasis (Oguri et al., 2024; Liu et al., 2020; Xiao et al., 2025; Ahn et al., 2025; Lavelle and Sokol, 2024). Among these, Lachnospiraceae, Ruminococcaceae, Bacteroides, and Lactobacillus are major producers of SCFAs and tryptophan metabolites, serving as the core executing taxa for shared antifibrotic mechanisms (Louis et al., 2022; Roager and Licht, 2018); profibrotic taxa drive fibrotic progression mainly by disrupting intestinal barrier integrity, disturbing bile acid metabolism and releasing proinflammatory mediators (Saha et al., 2025; Lavelle and Sokol, 2024; Yu et al., 2025). This review aims to clarify the “commonality–specificity” regulatory network of the gut microbiota in multiorgan fibrosis, thereby offering new insights and a theoretical basis for early warning, targeted intervention, and clinical translation in multiorgan fibrosis.

### 1.1 Literature search strategy

This manuscript is intended as a narrative review rather than a systematic review; therefore, no formal PRISMA-compliant search protocol or risk-of-bias assessment was performed. A structured comprehensive literature search was implemented across three authoritative electronic databases: PubMed, Web of Science, and Embase. The search window covered all publications from database inception up to June 2026. A combined retrieval strategy integrating MeSH subject headings and free-text terms was adopted. The core retrieval themes included the gut microbiota, intestinal flora, microbial metabolites (short-chain fatty acids, trimethylamine N-oxide, bile acids, tryptophan derivatives), gut–organ axes, and all subtypes of fibrotic disorders—hepatic, biliary, intestinal, pulmonary, myocardial, and cutaneous.

The inclusion criteria were peer-reviewed English publications, consisting of original *in vitro* cell and animal intervention experiments, multi-omics analyses, human cross-sectional/longitudinal cohort studies, Mendelian randomization causal inference analyses, meta-analyses, and authoritative mechanistic review articles. The exclusion criteria were as follows: conference abstracts, isolated case reports, editorial letters without systematic mechanistic elaboration, records without full-text access, duplicate entries retrieved across multiple databases, and articles irrelevant to the gut microbiota–fibrosis regulatory network.

Two independent researchers completed sequential screening by title, abstract, and full text. Any discrepancies in article eligibility were resolved via group discussion until a unified consensus was reached. A total of 117 eligible references were ultimately included. All included evidence was categorized according to affected organs, core microbial metabolites, and evidence grades: *in vitro* cellular data, animal causal intervention evidence, human associative cohort data, and genetic inference from Mendelian randomization. On the basis of the sorted evidence library, we constructed a unified dual-layer theoretical framework of shared core pathways vs. organ-specific differentiation to interpret divergent organ responses to identical microbial metabolites and to systematically summarize unresolved mechanistic conflicts and translational bottlenecks in this research field.

## 2 Gut–multiorgan axis: the systemic signaling network of microbial signals

Understanding the anatomical and physiological differences among various gut–organ axes in terms of signal carriers, transmission pathways, and target cell responses is a necessary prerequisite for establishing the dual-layer regulatory framework of common pathways vs. organ-specific pathways that underpins this review (Lin et al., 2025). Gut microbial signals are primarily delivered systemically through three routes: small-molecule metabolites are absorbed across the intestinal epithelium into the portal circulation and lymphatic system; microbial components undergo systemic translocation upon intestinal barrier disruption (Saha et al., 2025; Oami et al., 2025); and gut-activated immune cells migrate over long distances via the lymphatic system (Dang and Marsland, 2019; Xie et al., 2024). These three transmission modes are mutually coupled and together constitute the backbone of the global microbial signal transmission network.

The gut–liver axis possesses a unique bidirectional circulatory anatomy: 75% of the hepatic blood supply derives from the portal vein and continuously receives gut-derived metabolites and microbial molecules, whereas bile acids synthesized by the liver return to the intestine, reshaping the gut microbial composition in a reciprocal manner (Tripathi et al., 2018; Albillos et al., 2020). The gut–lung axis relies primarily on humoral circulation as its medium; enteric pathogens and metabolites can reach the lungs via the lymphatic route, recruiting γδT-17 cells to secrete IL-17 and amplify local inflammation (Budden et al., 2017; Yang J. et al., 2025). The gut–skin axis achieves distal signal delivery through the systemic circulation, where gut-derived metabolites modulate dermal fibroblast function; fecal microbiota transplantation and multiomics data have both confirmed their direct involvement in cutaneous matrix remodeling (O'Neill et al., 2016; Salem et al., 2018). The gut–heart axis utilizes circulating metabolites as core messengers, with TMAO and short-chain fatty acids acting directly on cardiac interstitial cells (Tang et al., 2017), but the microbiota may also indirectly aggravate cardiac injury by reshaping the systemic immune microenvironment (Wang J. et al., 2025). Beyond these distal trans-organ communications, two local regulatory pathways exist: intestinal metabolites act directly on resident gut cells to regulate local autocrine/paracrine activity, and the gut–spleen axis, as a systemic immune hub, receives portal microbial signals and activates proinflammatory immune cells, which then enter the circulation and infiltrate various organs to exacerbate fibrotic lesions (Dey, 2025; Seki and Schnabl, 2012; Li et al., 2017). Although the gut–brain axis can modulate systemic stress and inflammation via the vagus nerve and the HPA axis, there is currently no direct causal evidence for its involvement in multiorgan fibrosis; thus, it is not discussed in depth in this review (Stilling et al., 2016).

**Figure 1:**
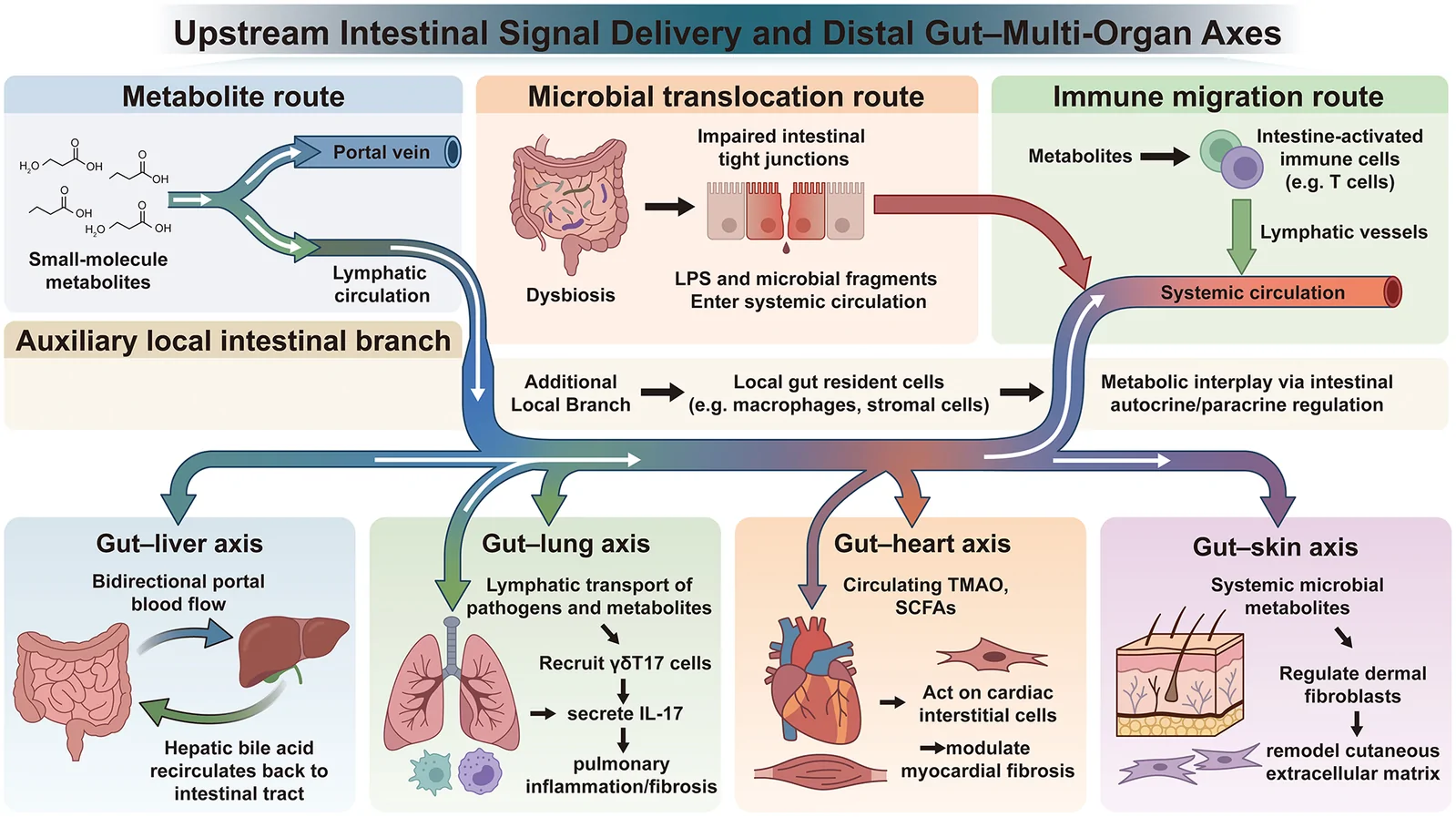
Upstream intestinal signal delivery and distal gut–multi-organ axes. Three major transport routes enable gut-derived signaling molecules to spread to peripheral tissues: metabolite trafficking via the portal vein and lymphatic circulation, microbial component translocation induced by dysbiosis and impaired intestinal tight junctions, and lymphocyte migration through lymphatic vessels. An auxiliary local intestinal branch mediates autocrine and paracrine crosstalk among gut-resident cells. Representative gut–liver, gut–lung, gut–heart, and gut–skin axes are summarized to illustrate how circulating gut signals regulate distal tissue homeostasis and fibrotic progression. Local intestinal fibrotic responses and the gut–biliary axis are further elaborated in Figure 3.

Metabolite transport and immune cell migration are not independent of each other: short-chain fatty acids, bile acids, and tryptophan derivatives can not only act directly on distal organs via the circulation but also preshape the metabolic phenotype of intestinal immune cells, generating preprogrammed immune cells capable of the targeted release of inflammatory mediators (Nogal et al., 2021; Lavelle and Sokol, 2024; Roager and Licht, 2018). This coupled metabolic–immune transmission system provides an anatomical-level explanation for the differential effects of the same metabolites across different organs. This chapter outlines only the anatomical network for the systemic transmission of microbial signals; the four core molecular mechanisms are systematically elaborated upon in Chapter 3, and organ-specific regulatory pathways are discussed in separate sections in Chapter 4. Gut microbial signals propagate to peripheral organs via metabolite circulation, microbial translocation and immune cell trafficking, forming the foundational gut–multi-organ signaling network illustrated in Figure 1.

## 3 Microbiota-derived metabolites in fibrotic processes: shared mechanisms

Relying on the gut–multiorgan axis signaling network, the gut microbiota and its metabolites regulate systemic multiorgan fibrosis through four major shared mechanisms (Lin et al., 2025; Lavelle and Sokol, 2024). However, these four pathways are not equally important across all organs; rather, their dominance is strictly determined by the target organ's anatomical position, receptor expression profile, and direct vs. indirect exposure to microbial products (Tripathi et al., 2018; Seki and Schnabl, 2012). Table 1 summarizes the organ-specific weighting of each mechanism based on current causal evidence: The bile acid/FXR pathway is liver- and biliary-dominant (direct portal vein exposure) and has weak or negligible direct effects on the myocardium, lung, and skin (which lack FXR/TGR5 expression) (Shi et al., 2023; Xiao et al., 2025). The TMAO pathway is heart- and skin-predominant, as these organs are primary targets of circulating TMAO but lack significant bile acid signaling (Kim et al., 2022; Wang J. et al., 2025). The SCFA/HDAC pathway is broadly conserved across all organs but acts through different dominant mechanisms (GPCR-mediated in immune cells vs. HDAC inhibition in parenchymal cells) (Nogal et al., 2021; Inamoto et al., 2023). The AhR pathway has the highest ligand- and cell-type specificity, making its organ-specific effects highly context dependent and often contradictory (Pan et al., 2025). This hierarchical framework guides the organ-specific discussions in Section 4, where we prioritize the dominant pathway for each organ and critically assess the relative contributions of secondary pathways.

**Table 1:** Cross-organ effects of major microbial metabolites in fibrosis.

Metabolite class	Metabolite (producer)	Target organ	Effect	Key pathways/mechanisms	Evidence strength	References
SCFAs	Propionate (Akkermansia muciniphila)	Liver	Anti-fibrotic	Activates Keap1–Nrf2 antioxidant pathway; suppresses TGF-β/SMAD-mediated hepatic stellate cell activation	Animal causal	Zhang et al., 2025
	Butyrate (Multiple genera)	Biliary tract	Anti-fibrotic	Activates PPARD/fatty acid β-oxidation to promote MDSC expansion; inhibits HDAC3 to block IL-6/STAT3 fibrotic signaling	Animal causal	Wang R. et al., 2024
	Butyrate (Multiple genera)	Small intestine	Anti-fibrotic	Downregulates myofibroblast markers α-SMA and collagen I; suppresses extracellular matrix deposition	Human correlational	Jurickova et al., 2022
	Butyrate (Multiple genera)	Heart	Anti-fibrotic	Inhibits HDAC activity to improve post-MI tissue repair (high-dose effects remain unclear)	Animal causal	Song et al., 2021
	Butyrate (Multiple genera)	Lung	Anti-fibrotic	Inhibits YBX1 ubiquitination; blocks alveolar EMT through CYP1A1/NF-κB signaling	Animal causal	Li et al., 2025
Choline metabolites	TMAO (gut-derived TMA; host FMO3 conversion)	Heart	Pro-fibrotic	Activates cardiac JAK2/STAT3 signaling; upregulates collagen and fibronectin synthesis	Animal causal	Seki and Schnabl, 2012
	TMAO (gut-derived TMA; host FMO3 conversion)	Skin	Pro-fibrotic	Activates PERK signaling; induces dermal fibroblast-to-myofibroblast conversion	Animal causal	Kim et al., 2022
Bile acids	Secondary bile acids	Liver	Bidirectional	Commensal bacteria regulate the bile acid pool; activate intestinal FXR–FGF15/19 to suppress hepatic CYP7A1 and reduce de novo bile acid synthesis	Animal + human MR	Xiao et al., 2025; Pandey et al., 2025
	Secondary bile acids (Pediococcus pentosaceus Li05)	Biliary tract	Bidirectional	Activates both hepatic FXR–SHP and intestinal FXR–FGF15 pathways to reduce bile acid accumulation; dysregulated bile acids exacerbate biliary injury	Animal causal	Fousekis et al., 2025; Han et al., 2023
Tryptophan metabolites	L-Tryptophan	Lung	Pro-fibrotic	Gut L-tryptophan accumulation activates mTOR/S6 signaling, synergizing with TGF-β1 to drive alveolar EMT	Animal causal	Li J. et al., 2024

This table summarizes key microbial metabolites and their reported organ-specific effects on fibrosis. The “Evidence Strength” column categorizes supporting evidence into three tiers based on study design and causal inference, consistent with the framework described in Section 6.2: Animal causal — *in vivo* intervention experiments (e.g., germ-free colonization, fecal microbiota transplantation, single-strain administration or metabolite reconstitution) that demonstrate causal regulatory effects; Animal + human MR — causal evidence from animal studies combined with human Mendelian randomization analyses indicating potential causal relationships; Human correlational — cross-sectional or retrospective cohort studies reporting statistical associations without causal verification. The “Key Pathways/Mechanisms” column incorporates relevant information regarding functional receptors, responder cell types and perturbed signaling cascades. Refer to Table 2 for organ-specific ranking of pathway predominance.

Existing animal intervention experiments—including germ-free mouse colonization, metabolite reconstitution, and gene knockout studies—have provided reliable experimental evidence for the causal regulatory roles of these pathways (Zhang et al., 2025; Zhao et al., 2023; Wang et al., 2022). Human cross-sectional and cohort studies, however, can only observe statistical associations between metabolite levels and fibrosis severity; owing to confounding factors, such human data alone cannot be used to establish causal pathogenic relationships (Loomba et al., 2017; Stec et al., 2023; Li J. et al., 2024). This section outlines the general regulatory logic of each pathway. The differential responses of different organs—owing to variations in cellular composition, receptor expression, and local microenvironments—will be elaborated in detail in Section 4 on an organ-by-organ basis.

### 3.1 Short-chain fatty acids: conserved antifibrotic mediators

Short-chain fatty acids (SCFAs), including butyrate, propionate, and acetate, are generated by the fermentation of dietary fiber by common commensal taxa (Louis and Flint, 2017). Cellular and animal intervention studies have confirmed that SCFAs directly inhibit fibroblast activation and collagen deposition through two core mechanisms: (1) activation of GPR41 and GPR43, which blocks the profibrotic TGF-β/Smad and NF-κB pathways (Trompette et al., 2014); and (2) acting as endogenous inhibitors of histone deacetylases (HDACs), which directly repress the transcription of profibrotic genes via epigenetic regulation, an effect independent of GPCR activation (Inamoto et al., 2023). Additionally, SCFAs maintain intestinal epithelial tight junctions, reduce gut permeability, and alleviate systemic low-grade inflammation (Saha et al., 2025). The reconstitution of SCFA-producing strains or exogenous SCFA supplementation in germ-free mice consistently reproduces the organ-protective phenotype, clearly establishing a causal antifibrotic role for SCFAs (Zhang et al., 2025; Oguri et al., 2024).

Human observational data generally reveal decreased levels of fecal SCFAs in fibrosis patients, which are negatively correlated with disease severity (Nogal et al., 2021); however, these associations are confounded by diet, medications, and sampling conditions. To date, no large-scale randomized controlled trials of oral SCFA supplementation or high-fiber dietary intervention for fibrosis have been conducted, and the protective effects in humans await clinical validation.

### 3.2 Tryptophan metabolites and the ahr signaling pathway: an immune-fibrosis regulatory hub with ligand-dependent plasticity

The aryl hydrocarbon receptor (AhR) pathway exemplifies the complexity of microbiota–host signaling in fibrosis, as its net effect is determined by a triple-layer regulatory logic: ligand identity, signaling mode, and cellular context (Pan et al., 2025). Understanding this hierarchy is essential for interpreting the seemingly contradictory organ-specific outcomes discussed in Section 4.

Ligand specificity is the primary determinant. AhR is a promiscuous receptor that binds structurally diverse ligands, triggering distinct conformational changes and coregulator recruitment that lead to entirely different transcriptional programs (Roager and Licht, 2018). Endogenous ligands derived from tryptophan metabolism—such as indole and indole-3-propionic acid produced by Lactobacillus and Bacteroides—generally favor anti-inflammatory outcomes by promoting Th17/Treg balance and suppressing IL-6, IL-17, and TNF-α (Yuan et al., 2025). In contrast, upon binding to AhR, exogenous ligands such as the environmental pollutant TCDD upregulate Snai2, downregulate E-cadherin and occludin, induce epithelial–mesenchymal transition, and exacerbate fibrosis (Pierre et al., 2014). Dietary indole-3-carbinol, another exogenous ligand, alleviates alcoholic liver injury, demonstrating that even structurally related exogenous ligands can produce opposing effects. Endogenous ITE blocks myofibroblast activation by inhibiting Smad2/3 nuclear translocation (Lehmann et al., 2011). This ligand-dependent plasticity means that simply targeting “AhR activation” is mechanistically meaningless; therapeutic strategies must specify the ligand or the specific transcriptional outcome desired.

The AhR pathway operates through two distinct signaling modes: canonical vs. noncanonical signaling pathways (Pan et al., 2025). In the canonical pathway, the ligand–AhR complex dimerizes with ARNT, translocates to the nucleus, and binds to DRE elements to regulate CYP family metabolic genes—a pathway traditionally associated with xenobiotic metabolism but increasingly recognized for its immunomodulatory functions. The noncanonical pathway mediates the interaction between AhR and NF-κB, ERK, and STAT3 through protein–protein interactions independent of DNA binding (Carter et al., 2025). This noncanonical mode enables AhR to modulate inflammatory signaling without altering gene transcription directly. For example, the TLR9–TDO2–AhR noncanonical axis in dendritic cells promotes IL-6 and IL-17 secretion, driving pulmonary fibrosis (Carter et al., 2025), whereas canonical AhR signaling in macrophages and myeloid cells tends to be anti-inflammatory and protective. The balance between these two modes—and whether they cooperate or compete—remains poorly understood but likely determines tissue-specific outcomes.

Cell-type specificity is the final layer of regulation. AhR effects are exquisitely cell type–dependent, reflecting the diverse expression of AhR interactors and downstream effectors across cell populations (Graelmann et al., 2024). Myeloid AhR activation alleviates high-fat–induced liver injury, whereas myeloid-specific knockout exacerbates steatofibrosis—a reversal that highlights the protective role of AhR in macrophages (Graelmann et al., 2024). AhR in hepatic stellate cells can selectively induce ferroptosis to protect hepatocytes, a mechanism not observed in other cell types (Liu et al., 2024). Renal tubular AhR inhibits NF-κB to reduce injury, whereas uremic endogenous ligands activate the same receptor to promote fibrosis—a ligand-dependent switch within the same cell type. Cardiac AhR deficiency amplifies angiotensin II-mediated fibrosis through the c-Jun/HIF-1α pathway, suggesting that in the heart, AhR is protective (Nakajima et al., 2019), whereas in other contexts, it may be deleterious. This cell-type specificity mandates that organ-level conclusions cannot be generalized; each tissue must be evaluated independently.

Current human studies have identified only statistical associations between AhR gene polymorphisms and fibrosis risk; no human ligand intervention trials have yet validated causal regulatory effects at the clinical level. Ligand diversity, pathway bifurcation, and cell-type specificity collectively establish AhR as a high-dimensional regulatory node whose manipulation requires precision far beyond that of SCFAs or bile acid modulators.

### 3.3 Intestinal barrier impairment and microbial translocation: profibrotic initiating triggers

Dysbiosis downregulates the expression of tight junction proteins, increasing intestinal permeability and allowing lipopolysaccharide (LPS) and bacterial nucleic acids to enter the bloodstream and persistently activate systemic TLR4/NF-κB inflammatory signaling, which in turn induces aberrant fibroblast activation in various organs (Saha et al., 2025; Awoniyi et al., 2023). LPS reconstitution and tight junction gene knockout animal models have confirmed that increased intestinal permeability causally promotes inflammation and fibrosis (Oami et al., 2025).

Clinical measurements revealed elevated plasma levels of LPS, zonulin, and other permeability markers in patients with fibrosis, and these levels were positively correlated with inflammation and the fibrotic stage (Loomba et al., 2017); however, these indicators are susceptible to interference from diet, medications, and assay methods: They can serve only as correlative clues and cannot independently establish increased intestinal permeability as an initiating cause of human fibrosis.

### 3.4 Bile acid metabolic dysregulation: a shared endocrine regulatory mechanism in fibrosis

The gut microbiota mediates the conversion of primary to secondary bile acids; dysbiosis leads to compositional disruption and abnormal accumulation of bile acids (Shi et al., 2023). Exogenous bile acid administration, FXR knockout, and fecal microbiota transplantation animal experiments have demonstrated that aberrant bile acid activity activates the TGF-β/Smad and NF-κB pathways through FXR and TGR5 receptors, driving fibroblast collagen synthesis (Xiao et al., 2025; Kasahara et al., 2023).

Cross-sectional human data show that bile acid profiles in patients with liver and biliary fibrosis differ significantly from those in healthy controls and that specific bile molecules are correlated with disease stage (Shi et al., 2023); however, confounding variables such as liver disease duration and cholagogic medications preclude the establishment of bile acid dysregulation as an independent causal factor.

### 3.5 Emerging mediators of microbiota–host communication in fibrosis

In addition to the four canonical metabolite pathways, several additional classes of mediators that expand the mechanistic repertoire of microbiota-driven fibrosis have been identified. These emerging pathways—those involving bacterial extracellular vesicles, sphingolipid metabolism, and metabolic reprogramming—are mechanistically distinct and operate through noncanonical signaling axes; however, they share the common feature of mediating transorgan communication without direct bacterial translocation (Lavelle and Sokol, 2024).

#### 3.5.1 Bacterial extracellular vesicles: nanoscale transorgan shuttles

Bacterial extracellular vesicles (BEVs) are nanoscale lipid bilayer particles secreted by both gram-negative and gram-positive bacteria, and they carry a cargo of LPS, nucleic acids, proteins, and metabolites. After intestinal barrier disruption, BEVs derived from pathogenic bacteria translocate to extraintestinal organs and activate fibrotic signaling via the TLR4, cGAS–STING, and TGF-β pathways in hepatic stellate cells and intestinal fibroblasts (Ahn et al., 2025). Conversely, probiotic-derived BEVs exert protective effects: vesicles from Lactobacillus amylovorus regulate the bile acid–FXR pathway to ameliorate cholestatic liver injury, whereas Faecalibacterium prausnitzii EVs induce macrophage M2b polarization to alleviate intestinal fibrosis (Wang Y. et al., 2024). The functional outcome of BEVs is determined primarily by the strain of origin—pathogenic vs. probiotic—rather than the cargo type alone. Engineered BEVs hold promise as organ-targeted delivery vehicles for antifibrotic agents. However, all BEV evidence is derived solely from cellular and animal experiments; human translocation, biodistribution, and functional relevance remain entirely unexplored.

#### 3.5.2 Sphingolipid metabolism: a lipid-mediated regulatory axis

Sphingolipids are emerging as key metabolic mediators at the microbiota–fibrosis interface. Ceramide, a central sphingolipid metabolite, activates NF-κB and TGF-β/Smad signaling through SMPD3-mediated production, driving MASH-associated fibrosis (Wang Q. et al., 2025). Neutral ceramidase disrupts intestinal immunity through the NcDase–SCD1–Wnt pathway. Mendelian randomization analyses suggest that plasma sphingomyelin is an intermediary variable between the gut microbiota and myocardial fibrosis, with a mediation effect of 14.2%, which is a statistical finding that has not yet been validated by interventional studies (Wang J. et al., 2025). Phytosphingosine, a component of F. prausnitzii vesicles, and microbiota-mediated glycosphingolipid modifications can both regulate macrophages and myeloid-derived suppressor cells, reshaping the immune microenvironment of fibrotic organs. Unlike SCFAs or bile acids, sphingolipid signaling operates through lipid-mediated receptor activation rather than through classical metabolite–receptor interactions, representing a distinct mechanistic class that may offer orthogonal therapeutic targets.

#### 3.5.3 Metabolic reprogramming: fibroblast energetics as a therapeutic node

Activated fibroblasts and inflammatory cells commonly undergo a metabolic switch to glycolysis-dominant energy production, supporting the extensive bioenergetic demands of collagen synthesis and cytokine secretion (Shang et al., 2025). Microbiota-derived LPS, TMAO, and acetaldehyde induce oxidative stress via the gut–liver axis, driving proinflammatory metabolic reprogramming in macrophages and epithelial cells. This reprogramming creates a feed-forward loop: glycolysis-derived lactate and succinate further stabilize HIF-1α, perpetuating inflammatory and fibrotic gene expression (Li Y. et al., 2026). In animal models, natural compounds such as kaempferol and pomegranate peel polyphenol extract have been shown to reshape gut microbiota homeostasis while simultaneously modulating arginine and proline metabolic pathways, thereby alleviating liver fibrosis and hepatic lipid accumulation (Zhang et al., 2026). However, these conclusions remain at the preclinical exploration stage; related targets may serve as auxiliary candidates for multitarget combination interventions rather than standalone therapies.

#### 3.5.4 Evidence hierarchy across emerging mediators

**Figure 2:**
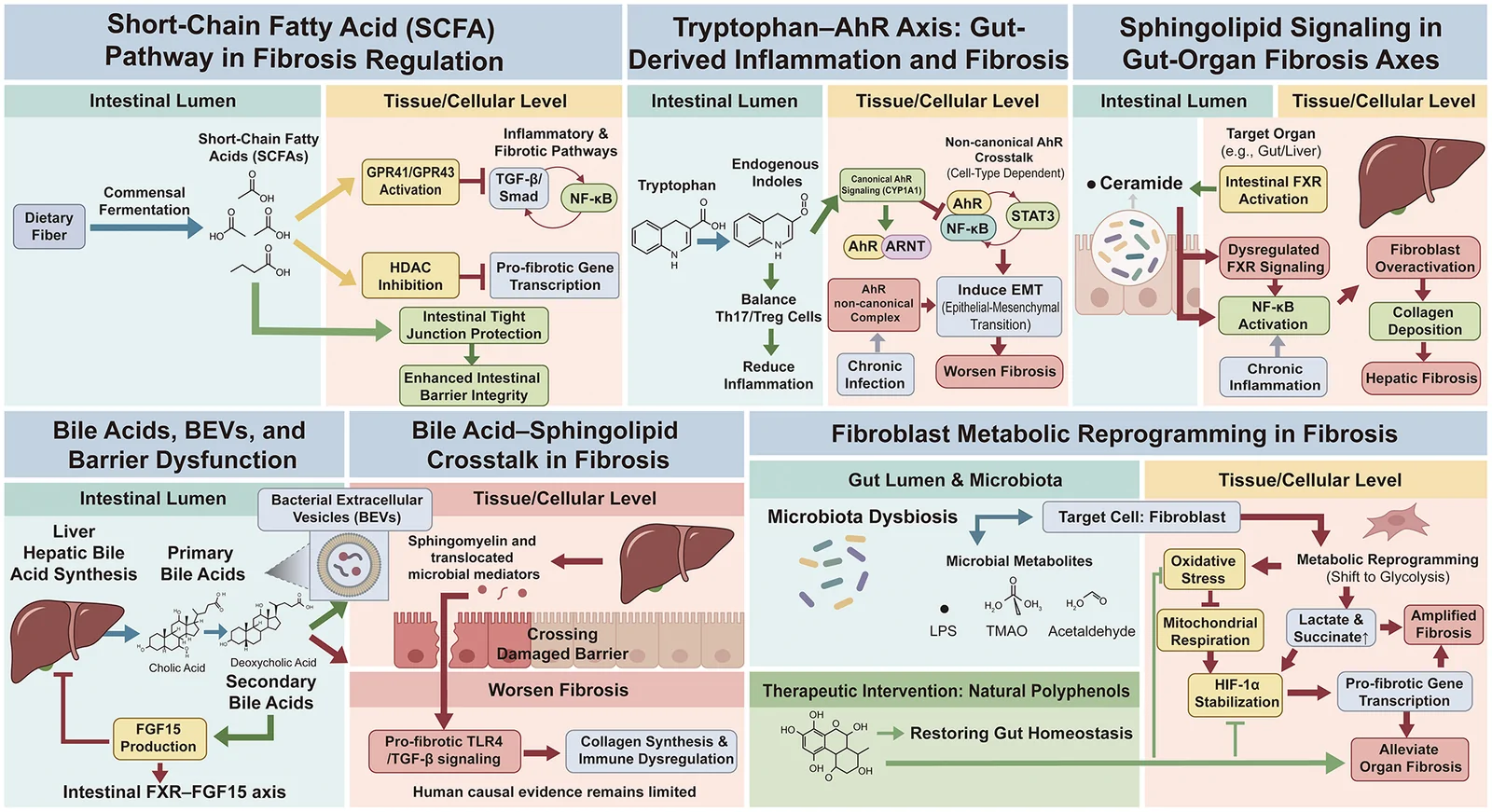
Core shared mechanistic pathways underlying gut microbiota-regulated organ fibrosis. This composite schematic summarizes six conserved signaling cascades mediating gut–organ fibrotic axes, including SCFA regulatory pathways, tryptophan–AhR signaling, sphingolipid signaling, bile acid–BEV barrier regulation, bile acid-sphingolipid crosstalk, and fibroblast metabolic reprogramming. Each module distinguishes events occurring within the intestinal lumen and downstream tissue/cellular responses. Canonical and non-canonical AhR signaling, intestinal barrier maintenance, FXR–FGF15 feedback axis, TLR4/TGF-β pro-fibrotic cascades, and glycolytic shift in fibroblasts are illustrated. Natural polyphenols are highlighted as candidate therapeutic agents to restore gut homeostasis and mitigate organ fibrosis. Note that causal evidence from human studies for bile acid-sphingolipid-mediated fibrotic signaling remains limited.

A critical evaluation of these emerging pathways reveals a stark evidence hierarchy. Exogenous butyrate and PEA administration provides complete antifibrotic causal evidence in animal models with pathway-specific inhibitor validation (Zhang et al., 2025; Zhou et al., 2025); in humans, only plasma metabolite levels are significantly associated with fibrosis stage, with no oral metabolite intervention RCTs conducted to date (Stec et al., 2023; Li J. et al., 2024). BEV and sphingolipid pathways are supported by animal and cellular evidence for mechanistic plausibility but lack even human correlational data for most fibrosis types (Ahn et al., 2025; Wang Y. et al., 2024). Metabolic reprogramming targets are supported only by basic cellular experiments and early-stage animal models (Shang et al., 2025; Li Y. et al., 2026). The evidence hierarchy across these emerging mediators is summarized as a text-based overview above; the organ-specific pathway ranking is presented later in Table 2 (see Section 6.1). Conserved shared signaling cascades mediate gut microbiota-associated fibrosis across tissues. These core mechanistic pathways are summarized schematically in Figure 2.

**Table 2:** Relative importance ranking of the four shared mechanisms in organ fibrosis based on evidence strength.

Organ	Pathways	Evidence and intervention validation	Microbiota/metabolite changes	Effects	References
Liver	Bile acids/FXR (+++)	Animal + human MR	Altered secondary bile acid composition: T-βMCA↓, CDCA↑	CYP7A1↓, bile acid synthesis↓	Liu et al., 2020; Xiao et al., 2025
	SCFAs (++)	Animal causal	Akkermansia muciniphila↓ → propionate↓	Keap1–Nrf2↓, HSC activation↑	Zhang et al., 2025
	Barrier/TLR4 (++)	Animal causal	LPS↑ (translocation)	NLRP3/GSDMD pyroptosis↑	Che et al., 2026
Biliary tract	Bile acids/FXR (+++)	Animal + human MR	Bile acid pool↑	FXR–SHP↑, FXR–FGF15↑	Han et al., 2023
	SCFAs (+)	Human correlational	Butyrate-producing bacteria↓ (PBC); butyrate↓ (BA infants)	HDAC2↑ → IL-6/STAT3↑	Wang R. et al., 2024; Kramer et al., 2025
Heart	TMAO (+++)	Animal bidirectional causal	TMAO↑ (circulating); Bacteroides vulgatus↑; Segatella↑	JAK2/STAT3↑; Sucnr1↑; TLR4/NF-κB↑	Wang J. et al., 2025; Yang X. et al., 2025
	SCFAs (++)	Animal causal	Butyrate-producing taxa↓ → butyrate↓	HDAC inhibition↓, remodeling↑	Song et al., 2021
Lung	SCFAs (++)	Animal causal	Dietary fiber → reduced butyrate production	YBX1↑ → NF-κB↑, EMT↑	Li et al., 2025
	Tryptophan-AhR (++)	Animal causal	Lachnospiraceae, Allobaculum altered → L-tryptophan↑	mTOR/S6↑	Li J. et al., 2024
Skin	TMAO-PERK (++)	Animal causal	TMAO↑ (circulating)	PERK↑, myofibroblast transformation↑	Kim et al., 2022
	SCFAs (+)	Human correlational	Butyrate↓ (replenishable)	HDAC3 inhibition↑	Park et al., 2021

Pathways are ranked using a three-tier grading system (+++/++/+). The grading follows the same evidence classification as the “Evidence Strength” column in Table 1, which is defined in Section 6.2: +++ — “Animal + human MR” or “Animal bidirectional causal” evidence; ++ — “Animal causal” evidence; + — “Human correlational” evidence only. This ranking reflects the strength of currently available evidence and causal validation, rather than the absolute biological importance of the pathways.

## 4 Organ-specific regulatory roles of the gut microbiota and its metabolites in fibrosis

### 4.1 Liver fibrosis

The liver continuously receives gut microbial components and circulating metabolites via the portal vein, with hepatocytes and hepatic stellate cells (HSCs) serving as the core response targets (Tripathi et al., 2018; Seki and Schnabl, 2012). The microbiota-mediated regulation of liver fibrosis can be divided into profibrotic and antifibrotic signaling pathways (Che et al., 2026). Unlike other organs, the liver is unique in having a high-precision, noninvasive microbiota-based predictive model and faces the distinct translational bottleneck of extremely low engraftment efficiency for live biotherapeutic products (Loomba et al., 2017).

#### 4.1.1 Profibrotic microbial signals and molecular pathways

Profibrotic signals primarily originate from microbial translocation resulting from intestinal barrier disruption (Saha et al., 2025; Oami et al., 2025). Lipopolysaccharide (LPS) reaches the liver via the portal vein and activates the NLRP3 inflammasome in hepatocytes, initiating GSDMD-dependent pyroptosis (Che et al., 2026). Synbiotic preparations have been shown to reshape the gut microbiota, repair the intestinal barrier, block LPS translocation, inhibit hepatocyte pyroptosis, and delay fibrotic progression in animal models (Che et al., 2026). Colonization by specific pathogens can also directly amplify chronic hepatic inflammation and drive collagen deposition via synergistic activation of the NF-κB, STAT3, and MAPK pathways, as demonstrated in models of Helicobacter hepaticus and Enterobacteriaceae enrichment (Cao et al., 2020; Sun et al., 2026).

#### 4.1.2 Antifibrotic microbial metabolites and targeted regulation

Liver-specific protective mechanisms converge on three major pathways, each targeting a distinct hepatic cell type:

First, microbial metabolites directly inhibit HSC activation, the central initiating event in liver fibrogenesis (Rockey et al., 2015). Complete causal evidence supports a propionate-mediated regulatory axis: propionate produced by specific commensals activates the Keap1-Nrf2 antioxidant pathway in HSCs, blocks TGF-β/SMAD signaling, and inhibits collagen synthesis (Zhang et al., 2025). Other commensal-derived signals downregulate the expression of fibrotic marker genes such as TGFBR1 and COL1A1 or ameliorate cholestatic liver injury by increasing FXR signaling (Zhao et al., 2023; Kasahara et al., 2023).

Second, the intestinal FXR–FGF15 endocrine axis remotely regulates hepatic bile acid homeostasis (Xiao et al., 2025; Awoniyi et al., 2023). This pathway represents a unique long-distance regulatory mode in the liver, functioning without direct microbial contact. Specific commensals reshape intestinal bile acid composition, activating intestinal FXR–FGF15 signaling, which remotely suppresses hepatic CYP7A1 and reduces de novo bile acid synthesis, thereby alleviating bile acid-induced liver injury (Xiao et al., 2025; Awoniyi et al., 2023).

Third, microbial metabolites block hepatocyte pyroptosis. Specific butyrate-producing and bile acid-modulating commensals increase intestinal bile salt hydrolase activity, reduce hepatic toxic bile acid accumulation, stabilize mitochondrial membrane potential, and block caspase-11-mediated hepatocyte pyroptosis, curtailing the release of profibrotic inflammatory mediators at the source (Zhao et al., 2023; Wang R. et al., 2024).

Among the four shared mechanisms, the bile acid/FXR pathway and SCFA-mediated HSC inhibition constitute the dominant protective axes in the liver (Zhang et al., 2025; Xiao et al., 2025), whereas the AhR pathway plays a secondary, context-dependent role—its activation can be either protective or deleterious depending on the ligand, a duality not observed with SCFAs or FXR agonists (Pan et al., 2025; Liu et al., 2024). Furthermore, the TMAO pathway has no significant regulatory role in liver fibrosis, as hepatic FMOs facilitate rapid clearance of portal TMAO, limiting hepatic profibrotic TMAO signaling (Jang et al., 2024). This mechanistic hierarchy directly informs translational prioritization: Compared with AhR modulators or TMAO-targeting strategies, FXR agonists and specific commensal-based interventions have stronger preclinical rationales for liver fibrosis (Loomba et al., 2017; Chang et al., 2025).

#### 4.1.3 Clinical translation prospects

The liver is the only fibrotic organ for which a high-precision, noninvasive microbiota-based predictive model exists. A random forest model constructed from gut signature taxa achieved an AUC of 0.936 for discriminating moderate-to-advanced MASLD fibrosis, independent of conventional serum scores (Loomba et al., 2017). In terms of intervention, probiotic mixtures have improved only surrogate endpoints, with no liver biopsy-confirmed evidence of fibrosis retardation (Silva-Sperb et al., 2024; Oliveira et al., 2024). Given the 10–20-year disease course, long-term hard-endpoint RCTs are extremely difficult to implement; the field-specific solution is to use noninvasive elastography as the primary endpoint combined with dynamic microbiota monitoring in preventive cohorts over ≥5 years (Silva-Sperb et al., 2024; Abd El Hamid et al., 2024). In contrast to the situation in other organs, the core translational bottleneck for liver microbiota lies in the extremely low engraftment efficiency of live biotherapeutic products in humans, making the improvement of strain retention a unique technical challenge (Pandey et al., 2025; Schnabl et al., 2025; Cerrito et al., 2025).

### 4.2 Biliary fibrosis

The core pathological features of biliary fibrosis are excessive collagen deposition in the bile duct wall and progressive luminal stricture. The bile acid/FXR pathway serves as the central regulatory axis (Shi et al., 2023; Awoniyi et al., 2023). Unlike fibrosis in other organs, biliary fibrosis is characterized by bacterial antigen molecular mimicry as an autoimmune initiation mechanism (Bogdanos et al., 2001; Nie et al., 2025) and is a specific predictor of drug efficacy in UDCA nonresponders (Wang R. et al., 2024; Lammert et al., 2021).

#### 4.2.1 Profibrotic microbial signals and molecular pathways

Biliary fibrosis is driven by three classes of microbiota-related mechanisms that act in concert: gut microecological dysbiosis, the translocation of pathogenic bacteria, and molecular mimicry mediated by commensal antigens (Awoniyi et al., 2023; Nie et al., 2025; Little et al., 2020). Dysbiotic microbiota disrupt systemic bile acid metabolism; abnormally accumulated bile acids continuously bind to FXR and TGR5 receptors on biliary epithelial cells, releasing sustained profibrotic signals (Shi et al., 2023; Fousekis et al., 2025). Pathogenic translocation further amplifies biliary fibrotic damage: specifically, translocated bacteria increase intestinal bile acid reabsorption, downregulate the expression of tight junction proteins, and exacerbate cholestasis-induced liver fibrosis (Saha et al., 2025; Liu M. et al., 2025). Molecular mimicry is a biliary-specific autoimmune initiation mechanism—gut commensals express antigenic epitopes homologous to human mitochondrial PDC-E2, triggering bile duct–specific autoimmune injury through cross-reactive immune responses (Bogdanos et al., 2001). This mechanism is particularly critical in PBC fibrosis (Nie et al., 2025) and is involved in the persistent progression of chronic biliary inflammation in patients with PSC (Awoniyi et al., 2023; Little et al., 2020).

#### 4.2.2 Antifibrotic microbial metabolites and targeted regulation

Short-chain fatty acids, especially butyrate, are key protective metabolites in biliary fibrosis (Wang R. et al., 2024; Lammert et al., 2021). Specific commensals simultaneously activate both hepatic FXR–SHP and ileal FXR–FGF15 pathways, reducing de novo bile acid synthesis and alleviating cholestatic injury (Xiao et al., 2025; Han et al., 2023). Butyrate achieves antifibrotic activity through two independent pathways: in PBC, it activates PPARD-dependent fatty acid β-oxidation, promoting MDSC proliferation and immunosuppressive function, thereby alleviating biliary autoimmune injury (Wang R. et al., 2024); in biliary atresia, it inhibits HDAC2, blocks IL-6/STAT3 activation, and directly suppresses HSC activation and collagen deposition (Kramer et al., 2025).

#### 4.2.3 Clinical translation prospects

Translational efforts in biliary fibrosis leverage the unique clinical unmet need in autoimmune cholangiopathies: PBC and PSC have only UDCA as a first-line drug (Nie et al., 2025; Little et al., 2020), and fecal butyrate is the only field-specific predictor of drug efficacy, with significantly lower concentrations in UDCA nonresponders (Wang R. et al., 2024; Lammert et al., 2021). Leveraging the molecular mimicry mechanism, biliary fibrosis offers a unique approach for ultra-early screening: antimitochondrial antibodies can be detected years before clinical PBC onset, potentially enabling prefibrotic risk warning (Bogdanos et al., 2001). In PSC patients with concurrent IBD, the risk of colorectal cancer is significantly elevated, and monitoring the microbiota could simultaneously predict biliary fibrosis and intestinal neoplasia—a dual-screening value not found in other organs (Awoniyi et al., 2023; Little et al., 2020; Fousekis et al., 2025).

### 4.3 Intestinal fibrosis

The intestinal mucosa is in continuous direct contact with the luminal microbiota and its metabolites (Mai et al., 2025; Li X. et al., 2024). Importantly, the role of SCFAs in intestinal fibrosis presents a paradoxical contradiction that is rarely discussed in the literature: while SCFAs are universally considered antifibrotic on the basis of *in vitro* and systemic administration studies (Nogal et al., 2021; Jurickova et al., 2022), butyrate has been shown to exacerbate colonic inflammation in certain IBD contexts by fueling epithelial metabolism and increasing IL-1β secretion (Li X. et al., 2024), suggesting that SCFA-based interventions could be detrimental to active colonic fibrosis. This contradictory evidence highlights a critical gap—most studies use global SCFA supplementation rather than organ-targeted delivery, and the net effect likely depends on baseline inflammatory status, luminal SCFA concentration gradients, and the differential responsiveness of colonic vs. small intestinal fibroblasts (Lavelle and Sokol, 2024).

Critically, the driving patterns of microbiota-mediated fibrosis regulation differ fundamentally between the small intestine and the colon, representing two distinct regulatory logics—inflammation-driven (colon) vs. metabolism-protective (small intestine) (Li X. et al., 2024; Jurickova et al., 2022). This dichotomy is not merely descriptive but reflects the dominance of different mechanisms: In the colon, the AhR/TLR4 inflammatory axis and purine metabolite–driven NLRP3 activation overwhelm the protective effects of SCFAs (Ahn et al., 2025; Mai et al., 2025); in the small intestine, where luminal SCFA concentrations are naturally higher and inflammatory stimuli are lower, the SCFA/HDAC pathway takes precedence (Nogal et al., 2021; Jurickova et al., 2022). Thus, any microbiota-based therapeutic strategy for intestinal fibrosis must be site specific: Small intestinal fibrosis may benefit from butyrate supplementation, whereas colonic fibrosis may require pathogen-targeted approaches rather than generalized SCFA elevation (Li X. et al., 2024).

#### 4.3.1 Colonic fibrosis: inflammation-driven

Colonic fibrotic progression is driven by the synergistic action of pathogenic bacterial invasion, purine metabolism disturbance, and overall microecological imbalance. A key proinflammatory strain invades the colonic mucosa and persistently induces local chronic inflammation (Ahn et al., 2025). The mechanism involves the secretion of a siderophore that chelates intestinal zinc, stabilizing HIF-1α in macrophages and reprogramming them toward a profibrotic phenotype (Ahn et al., 2025; Dehantschutter and Taylor, 2025). Moreover, purine metabolic remodeling is an important regulatory node: Decreased abundance of uric acid-degrading taxa and enrichment of uric acid-producing taxa lead to uric acid accumulation, which activates the NLRP3 inflammasome in intestinal epithelial cells, amplifying local inflammation and indirectly promoting fibroblast activation (Mai et al., 2025; Di Petrillo et al., 2025). Clinical cohorts have shown significantly elevated serum uric acid/creatinine ratios in IBD patients, which are positively correlated with stricture risk (Mai et al., 2025; Gu et al., 2025).

#### 4.3.2 Small intestinal fibrosis: metabolism-protective

Small intestinal fibrosis is most commonly seen in patients with Crohn's disease (Li X. et al., 2024). Unlike the inflammation-dominant colonic pattern, the regulation of fibrosis in the small intestine focuses on the protective effects of microbial metabolites. Microbiota-derived butyrate is a core protective metabolite: In organoid models of stenotic Crohn's disease tissue, butyrate downregulates the expression of myofibroblast markers and suppresses the expression of genes related to stricture-associated ECM synthesis (Jurickova et al., 2022); *in vitro* experiments have verified that butyrate and propionate induce intestinal fibroblast quiescence or apoptosis (Nogal et al., 2021; Jurickova et al., 2022). At the functional level, specific butyrate-producing commensals induce macrophage polarization toward the antifibrotic M2b phenotype and attenuate inflammatory fibrosis by balancing the Th17/Treg ratio (Wang Y. et al., 2024; Park et al., 2018). Notably, butyrate-producing taxa are significantly depleted in strictured mucosa, and their abundance is inversely correlated with postoperative recurrence risk, suggesting that this functional capacity is a potential predictor of surgical outcomes (Li X. et al., 2024; Park et al., 2026).

#### 4.3.3 Clinical translation prospects

Intestinal fibrosis is associated with a unique surgical clinical positioning: Intestinal stricture in patients with Crohn's disease is a visible clinical endpoint, and the core value of microbiota-based biomarkers lies in predicting postoperative stricture recurrence and assisting in surgical timing decisions—a surgical guidance function not found in other organs (Li X. et al., 2024). Mucosal butyrate-producing capacity is an intestine-specific postoperative recurrence biomarker (Park et al., 2026). Intestinal fibrosis has a clear intervention boundary: Mature fibrotic strictures cannot be reversed, and probiotics can prevent stricture formation only in the preoperative inflammatory phase (Park et al., 2018, 2026). The uric acid/creatinine ratio could be incorporated into a composite stricture prediction model but has insufficient specificity as a standalone tool (Mai et al., 2025; Gu et al., 2025).

### 4.4 Pulmonary fibrosis

The lung is unique in being subject to triple regulation from microbiota-related sources: gut-derived metabolites via the systemic circulation, oral pathogens inhaled via the respiratory tract, and locally colonizing microorganisms (Dang and Marsland, 2019; Budden et al., 2017; Yang J. et al., 2025). While these signals interweave, current evidence allows for a provisional hierarchical ranking: Gut-derived metabolites (especially PEA and SCFAs) exert the strongest causal antifibrotic effect, supported by multiple reconstitution studies and Mendelian randomization evidence (Zhou et al., 2025; Li et al., 2025; Fan et al., 2024); oral pathogens play a secondary, disease-modifying role that may accelerate progression but is unlikely to be sufficient as a sole driver (Ye et al., 2025); and lung-resident opportunists act as contextual amplifiers, predominantly in immunocompromised or postinfection settings (Hashem et al., 2025). This hierarchy has direct translational implications: Preventive strategies should prioritize gut-targeted metabolite elevation over oral hygiene interventions, which have shown inconsistent effects in IPF cohorts (Yang J. et al., 2025).

Importantly, the dominant mechanism varies by disease subtype—in systemic sclerosis-associated ILD, the gut–lung axis via PEA appears to be the primary driver (Zhou et al., 2025; Ni B. et al., 2025), whereas in idiopathic pulmonary fibrosis, genetic susceptibility likely overrides microbiota-derived signals, explaining why some microbiota-targeted interventions show modest effects in IPF but stronger effects in SSc-ILD (Fan et al., 2024; Luo et al., 2025). This subtype-specific hierarchy remains underappreciated in the literature and warrants dedicated head-to-head comparative studies.

Palmitoylethanolamide (PEA) is a lung-specific protective mediator; specific commensals reshape the gut microbiota, increase circulating PEA levels, and alleviate pulmonary fibrosis by inhibiting the TGF-β1/Smad2/3 pathway (Zhou et al., 2025). Furthermore, the lung is the only organ for which Mendelian randomization evidence supports a causal microbiota association (Fan et al., 2024).

#### 4.4.1 Profibrotic microbial signals and molecular pathways

Profibrotic signals in the lung originate from three distinct sources. In the gut-derived pathway, disturbed tryptophan-metabolizing capacity leads to L-tryptophan accumulation in lung tissue, which persistently activates the mTOR/S6 pathway and synergizes with TGF-β1 to promote alveolar epithelial–mesenchymal transition (Li J. et al., 2024; Roager and Licht, 2018). In the oral-derived pathway, periodontal pathogens invade lung tissue upon inhalation, inducing IL-17A-driven chronic inflammation and collagen deposition (Ye et al., 2025). Additionally, lung-resident opportunistic pathogens can elicit persistent local inflammation (Hashem et al., 2025).

#### 4.4.2 Antifibrotic microbial metabolites and targeted regulation

Multiple gut commensals exert pulmonary protective effects through independent signaling axes. Specific Bifidobacterium strains reshape the gut microecology, downregulate proinflammatory mediators, and balance PPAR signaling and Th17 responses (Ni J. et al., 2025). In the SCFA-related pathway, butyrate inhibits YBX1 ubiquitination and blocks alveolar epithelial–mesenchymal transition through the CYP1A1/NF-κB pathway (Li et al., 2025); specific butyrate-enriched strains upregulate pulmonary SCFA receptors, suppress macrophage proinflammatory activation and fibroblast differentiation, and ameliorate systemic sclerosis-associated pulmonary fibrosis (Park et al., 2024).

#### 4.4.3 Clinical translation prospects

Pulmonary fibrosis is the only organ for which Mendelian randomization genetic evidence supports microbiota associations: MR analyses have confirmed that butyrate-producing genera reduce the genetic risk of IPF (Fan et al., 2024). IPF patients tolerate lung biopsy poorly, making endpoint RCTs difficult; lung-specific surrogate markers include plasma KL-6, SP-D, and MMP-7 (Yang J. et al., 2025). PEA is a lung-specific dietary supplementable metabolic target, and preventive intervention trials are preferentially recommended in high-risk populations (Zhou et al., 2025). Oral pathogens serve as an independent screening dimension, leveraging upper–lower respiratory tract interactions as a unique supplemental risk assessment approach (Ye et al., 2025).

### 4.5 Myocardial fibrosis

Myocardial fibrosis relies on the gut–heart axis for remote regulation by circulating metabolites (Tang et al., 2017; Brown and Hazen, 2018). The myocardium has no direct contact with bile acids or luminal contents, making the TMAO–JAK2–STAT3 pathway a uniquely characteristic profibrotic axis distinct from the bile acid/FXR-dependent regulation seen in the hepatobiliary system (Yang X. et al., 2025; Zhu et al., 2016). These findings suggest that myocardial fibrosis is the most translationally mature microbiota-based biomarker, TMAO, which is already routinely used for prognostic stratification in heart failure patients (Tang et al., 2017; Zhu et al., 2016).

#### 4.5.1 Profibrotic microbial signals and molecular pathways

Profibrotic signals in the myocardium involve at least three independent pathways. First, gut-derived TMAO activates myocardial JAK2–STAT3 signaling, upregulating fibronectin and type I/III collagen expression (Yang X. et al., 2025). Second, microbiota-derived succinate induces fibrosis via specific receptor activation (Wang J. et al., 2025). Third, gut dysbiosis drives myocardial remodeling through the TLR4/NF-κB pathway (Graelmann et al., 2024). These three pathways—mediated by a circulating metabolite, a microbial secretion product, and inflammatory signaling—together constitute a unique multilayered profibrotic network in the heart.

#### 4.5.2 Antifibrotic microbial metabolites and targeted regulation

Butyrate-producing commensals and composite probiotics constitute the main protective system: Specific strains attenuate myocardial inflammation by stabilizing regulatory T cells (Wang et al., 2022), and butyrate-producing taxa inhibit HDACs and improve interstitial remodeling after infarction (Song et al., 2021). Other commensals generate bioactive metabolites that regulate mitochondrial dynamics to counteract fibrosis (Borshchev et al., 2025). Meta-analyses confirm that probiotics overall improve systemic low-grade inflammation in heart failure patients (Zhu et al., 2025).

#### 4.5.3 Clinical translation prospects

Myocardial fibrosis is associated with the most translationally mature biomarker, TMAO (Tang et al., 2017; Zhu et al., 2016). Studies in multicenter cardiovascular cohorts have confirmed its significant association with long-term prognosis, with minimal short-term dietary interference, and it is already routinely used for prognostic stratification (Tang et al., 2017). The cardiac-specific quantitative imaging parameter CMR ECV precisely quantifies interstitial collagen (Zhu et al., 2024); a field-adapted solution is to construct a composite score combining TMAO with established cardiac biomarkers. For intervention, TMAO synthesis inhibitors represent a unique small-molecule pipeline, pending only human safety trials (Zhu et al., 2016).

### 4.6 Skin fibrosis

Skin fibrosis exhibits a unique dual regulatory mode mediated jointly by circulating gut-derived metabolic signals and local mechanical signals (O'Neill et al., 2016; Kim et al., 2022; Liao et al., 2025). A critical but unanswered question is whether circulating microbial metabolites or local mechanical signals dominate in the development of skin fibrosis. Current evidence suggests a tissue-context–dependent hierarchy: In early-stage systemic sclerosis, the TMAO/PERK pathway may predominate, as plasma TMAO levels correlate with skin thickness scores independent of mechanical factors (Kim et al., 2022; Stec et al., 2023); however, in established keloids and localized scleroderma, local mechanical stiffness and Piezo1-mediated signaling likely override systemic microbial inputs, as these lesions progress independently of changes in the gut microbial composition (Liao et al., 2025). This distinction is not merely academic but dictates therapeutic strategy: Early SSc may benefit from systemic TMAO-lowering interventions, whereas keloids require local mechanical unloading or Piezo1 inhibitors, with microbiota modulation playing a negligible adjunctive role (Kim et al., 2022; Liao et al., 2025).

Furthermore, contradictory evidence exists regarding the role of TMAO in SSc: while cross-sectional studies have shown positive correlations, a recent longitudinal cohort study revealed no association between baseline TMAO levels and the 2-year progression of skin fibrosis, suggesting that TMAO may be a marker of disease activity rather than a causal driver (Stec et al., 2023). This methodological limitation—reliance on cross-sectional designs—undermines the translational enthusiasm for TMAO as a therapeutic target in skin fibrosis and should be prioritized for resolution through prospective serial sampling studies.

#### 4.6.1 Profibrotic microbial signals and molecular pathways

The TMAO-mediated PERK pathway is a core profibrotic signaling pathway that drives skin fibrosis (Kim et al., 2022). In animal models and SSc patient biopsies, TMAO activates PERK signaling, inducing fibroblasts and vascular cells to transform into myofibroblasts and drive dermal collagen deposition (Kim et al., 2022). Clinical studies have shown that plasma TMAO levels in SSc patients are significantly elevated and positively correlated with multiple organ complications, suggesting that it has potential as a serological monitoring indicator; however, most related studies are cross-sectional and lack longitudinal temporal data (Stec et al., 2023).

#### 4.6.2 Antifibrotic microbial metabolites and targeted regulation

Short-chain fatty acids, especially butyrate, are the major protective metabolites (Nogal et al., 2021; Park et al., 2021). Specific strains that enrich SCFA-producing taxa increase butyrate levels, upregulate SCFA receptor expression, and alleviate skin and pulmonary fibrosis (Shi et al., 2023; Park et al., 2021). Butyrate also inhibits HDAC activity, reprograms macrophage polarization, and attenuates the profibrotic response of human dermal fibroblasts to TGF-β1 (Inamoto et al., 2023; He et al., 2025). Plant-derived ursolic acid blocks TGF-β/Smad signaling to inhibit collagen synthesis while simultaneously activating MMP-1-mediated collagen degradation, bidirectionally regulating collagen homeostasis (He et al., 2025).

#### 4.6.3 Clinical translation prospects

Skin fibrosis has unique advantages for surface assessment: Sclerotic lesions can be visually inspected and biopsied, with the lowest follow-up costs (Kim et al., 2022). Its clinical translation involves two distinct pathways. SSc is a rare disease with strong subtype heterogeneity, precluding large-scale RCTs; the specific trial design should be single-arm open-label with self-controlled comparisons (Stec et al., 2023). Keloids, in contrast, have a large population base and measurable lesion volume changes within 3–6 months; the local intralesional injection of active compounds prevents systemic toxicity, making this a unique lead clinical model for local administration (Liao et al., 2025). The local mechanical target Piezo1–YAP is supported only by cellular/animal evidence (Liao et al., 2025). Overall, both biomarkers and intervention vectors for skin possess unique local-administration features.

**Figure 3:**
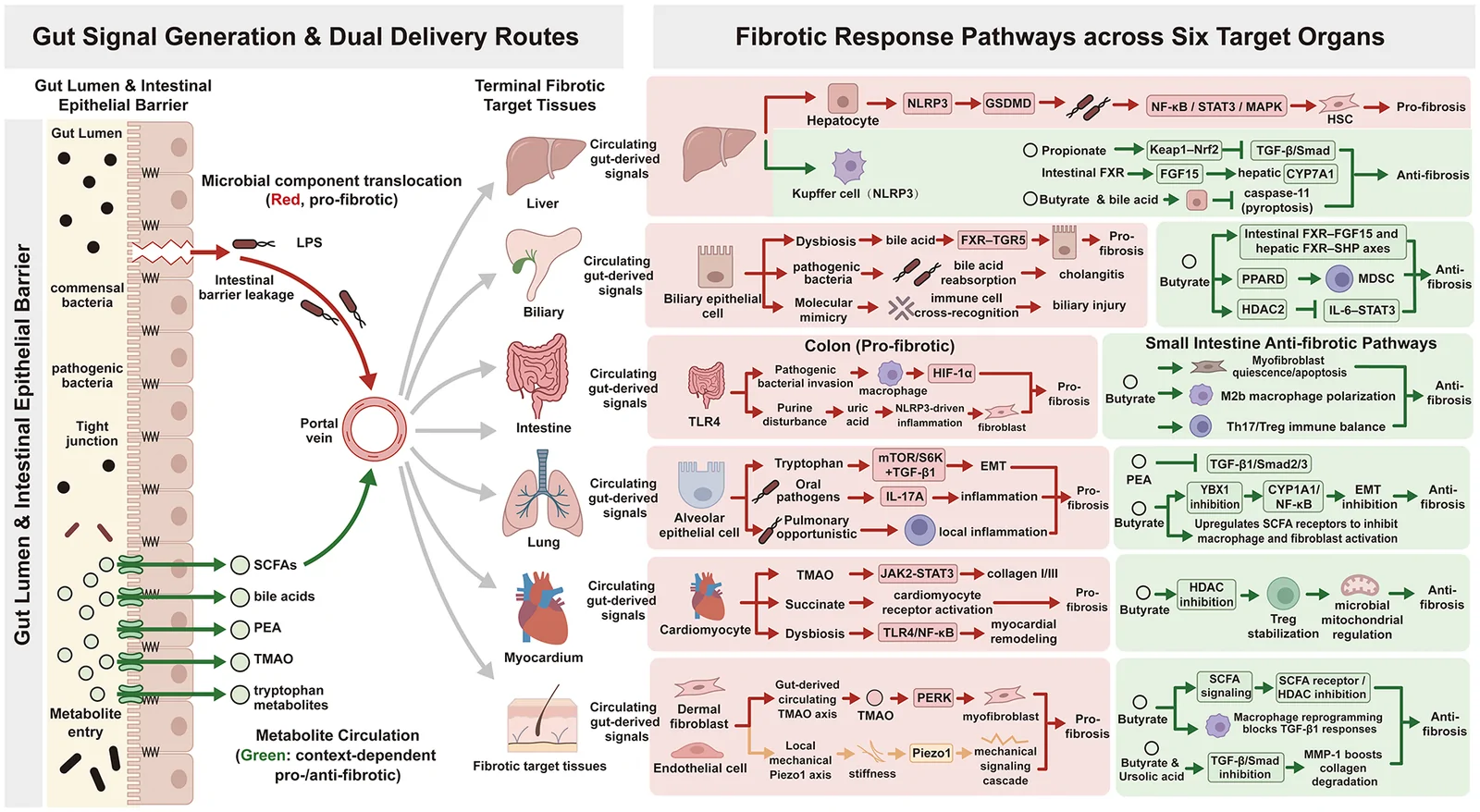
Gut signal generation and organ-specific fibrotic responses triggered by circulating gut-derived microbial signals. Schematic overview illustrating the generation of gut-derived signaling molecules and their systemic delivery via portal circulation. Disruption of the intestinal epithelial barrier facilitates translocation of microbial components (pro-fibrotic, labeled in red), while microbial metabolites (including SCFAs, bile acids, tryptophan metabolites, PEA and TMAO, labeled in green) enter systemic circulation. These circulating gut signals travel to six target organs (liver, biliary tract, intestine, lung, myocardium, and skin) and trigger divergent fibrotic phenotypes. For each organ, pro-fibrotic cascades and anti-fibrotic regulatory pathways mediated by distinct microbial metabolites are summarized, highlighting tissue-specific signaling axes, effector cell populations, and key molecular mediators.

The organ-specific microbial metabolite networks and signaling pathways across the liver, biliary tract, intestine, lung, myocardium, and skin are summarized in Table 1 for cross-organ comparison. Despite identical upstream gut microbial triggers, circulating metabolites and microbial components induce divergent fibrotic responses across distinct organs. These tissue-specific signaling pathways are illustrated in Figure 3.

## 5 Methodological limitations and future directions

### 5.1 Limitations at the study design level

A synthesis of the organ-specific evidence reveals several cross-cutting methodological limitations at the study design level that constrain the field's ability to establish causal hierarchies:

First, nearly all human data are cross-sectional, as metabolites and microbial composition are measured at a single time point. This design cannot distinguish whether microbial changes precede fibrosis (causal) or follow it (consequential) (Loomba et al., 2017; Stec et al., 2023). The only organ with prospective longitudinal data is the liver (via the 37-taxa prediction model), and this should be extended to other organs (Loomba et al., 2017).

Second, animal-to-human translation is confounded by fundamental differences in microbial ecology and metabolite kinetics: rodent SCFA production rates are 5–10-fold higher per unit body mass than those of humans are, and gut transit times differ substantially, potentially inflating the apparent protective effect of SCFAs in preclinical models (Nogal et al., 2021). This quantitative mismatch is rarely discussed in mechanistic papers.

Third, most organ-specific studies use single-mechanism interventions (e.g., butyrate alone or TMAO inhibition alone), but *in vivo*, these pathways operate in parallel and may exhibit synergistic or antagonistic interactions. For example, elevated TMAO may downregulate SCFA receptor expression in the heart, creating a profibrotic dominant state; however, such pathway cross-talk is almost entirely unexplored (Lavelle and Sokol, 2024).

Fourth, the emerging mediators discussed in Section 3.5—BEVs, sphingolipids, and metabolic reprogramming—remain at the earliest stages of evidence, with no human interventional data and limited human correlational data (Ahn et al., 2025; Wang Y. et al., 2024; Li Y. et al., 2026). Their inclusion in mechanistic models remains speculative and requires dedicated validation.

### 5.2 Experimental heterogeneity as a source of conflicting evidence

Beyond study design limitations, conflicting conclusions regarding the same microbiota or metabolite across studies often arise from experimental system heterogeneity rather than true mechanistic contradictions. These sources of heterogeneity can be categorized into three levels.

At the strain level, different strains within the same genus encode distinct metabolic enzyme repertoires—for example, some Lactobacillus strains synthesize tryptophan-derived indole ligands to activate AhR, whereas others lack this pathway (Liu A. et al., 2025). At the intervention level, short-term low-dose probiotic supplementation may restore intestinal permeability, whereas long-term high intake can disrupt bile acid homeostasis and induce injury (Craven et al., 2020). At the host level, the local microenvironment in advanced inflammation or fibrosis can suppress probiotic metabolic functions, whereas protective effects are fully manifested in healthy hosts (Lavelle and Sokol, 2024). The combination of these three conditions can produce completely opposite phenotypes for the same genus across different studies.

The same metabolite can exert divergent effects across different organs and models because of target organ molecular characteristics and administration conditions. TMAO exerts profibrotic effects only in the heart and skin, as hepatocytes highly express FMO oxidases that rapidly clear circulating TMAO, preventing the liver from mounting a comparable injury response (Kim et al., 2022; Yang X. et al., 2025; Silva-Sperb et al., 2024). SCFAs are generally protective, but their cardiac effects are bidirectionally variable: Low-dose butyrate inhibits HDAC activity and attenuates interstitial remodeling, whereas high-dose butyrate activates myocardial NF-κB proinflammatory signaling (Song et al., 2021); additionally, pressure-overload and chemical-injury heart failure models differ in microbial composition and cellular epigenetic backgrounds, further exacerbating experimental discrepancies (Kurban et al., 2025). The tryptophan–AhR pathway is bidirectionally regulated by ligand duration and intracellular cochaperone proteins: exogenous TCDD causes persistent activation that promotes fibrosis, whereas endogenous indoles activate only transiently (Pan et al., 2025; Pierre et al., 2014); the AhR–ARNT complex in macrophages favors anti-inflammatory responses, whereas the AhR–Src pathway in epithelial cells is predisposed to epithelial–mesenchymal transition, resulting in opposite effects of the same receptor in different systems (Carter et al., 2025; Graelmann et al., 2024).

Core differences among mainstream experimental systems introduce additional heterogeneity: 16S amplicon sequencing can identify taxa only at the genus level, whereas metagenomics can resolve strain-level functional genes, resulting in marked differences in taxonomic resolution (SYNCH Study Group, n.d.); live bacterial gavage vs. diet-induced metabolite production alters *in vivo* metabolite peak time and tissue exposure levels (Trompette et al., 2014); and chemical injury, bile duct ligation, and genetic knockout models each activate distinct core proinflammatory/profibrotic pathways that do not overlap (Sun et al., 2026). In addition, sample collection timing, microbial DNA extraction kits, and sequencing batch effects can all interfere with quantitative microbiota abundance results.

When interpreting and horizontally comparing data from multiple studies, the complete set of experimental conditions—including strain, dosage, model, and detection methods—must be fully considered (FARGO Study Group, n.d.). Future efforts should promote the establishment of standardized experimental protocols covering strain typing standards, metabolite quantification workflows, model selection principles, and comprehensive reporting of experimental parameters to reduce interstudy heterogeneity and enhance the comparability of results across studies.

To advance the field, we propose a mechanistic hierarchy validation framework that addresses both study design and experimental heterogeneity limitations: (1) multiomics time series in prospective cohorts to establish temporal directionality (Loomba et al., 2017); (2) pathway-specific inhibitor studies in human organoids to quantify relative effect sizes (FMT-NAFLD Study Group, n.d.); and (3) head-to-head intervention trials to compare dominant vs. secondary pathway modulators in the same organ. Until such studies are conducted, the hierarchies proposed in this review should be considered testable hypotheses rather than established paradigms.

## 6 Discussion

### 6.1 Organ-specific comparison of the four shared mechanisms

In contrast to the metabolite-centric horizontal comparison presented in Section 3.5.4, this section compares the relative contribution weights of the four transorgan shared mechanisms—SCFA signaling, the tryptophan–AhR pathway, intestinal barrier homeostasis, and bile acid/FXR signaling—across different organ fibrosis types from a pathway dominance perspective (Henderson et al., 2020; Lin et al., 2025). On the basis of the experimental and clinical evidence presented in the organ-specific subsections of Section 4, Table 2 ranks the relative contributions of the four pathways in each organ using a three-tier grading system (+++/++/+). Notably, this grading reflects the richness of currently published evidence and the strength of causal validation rather than the absolute biological importance of the pathways themselves (Loomba et al., 2017; Zhang et al., 2025; Sun et al., 2026).

The molecular basis for the organ-specific pathway rankings can be summarized by three determinant factors: ligand exposure mode (high-concentration portal delivery vs. systemic dilution vs. local *in situ* production), target cell receptor repertoire (differential expression of FXR, TGR5, GPCRs, TLRs, etc.), and metabolic clearance capacity (e.g., efficient hepatic FMO-mediated clearance of TMAO) (Tripathi et al., 2018; Kim et al., 2022; Silva-Sperb et al., 2024). Together, these three factors determine the organ distribution pattern of pathways graded as “+++” in Table 2 (see Table 2 for specific grading results).

Synthesizing the grading results, pathways ranked as “+++” can be prioritized as targets for organ-specific new drug development, whereas pathways marked only with “+” have weak evidence and are not suitable as standalone intervention directions (Chang et al., 2025; Lavelle and Sokol, 2024). From the perspective of broad-spectrum fibrosis prevention, the SCFA and tryptophan–AhR pathways exert regulatory effects across multiple organs and are therefore more suitable for the development of universal combination intervention strategies (Nogal et al., 2021; Pan et al., 2025).

### 6.2 Evidence limitations and causality

On the basis of study design, existing evidence can be classified into three tiers of strength: (1) direct causal evidence: germ-free mouse colonization, fecal microbiota transplantation, and single-strain/metabolite *in vivo* reconstitution interventions, which can bidirectionally validate causality through “phenotype recovery upon loss and lesion reappearance upon supplementation” (Zhang et al., 2025; Zhao et al., 2023; Wang et al., 2022); (2) weak causal indication: Mendelian randomization analysis can reduce confounding and reverse causation bias but remains statistical inference and cannot be equated with direct causal intervention-level evidence (Fan et al., 2024); and (3) purely associative evidence: cross-sectional, retrospective cohort, and multiomics association analyses can observe only correlations and cannot distinguish causal direction, with residual confounding difficult to fully exclude (Loomba et al., 2017; Stec et al., 2023).

Under this hierarchical standard, *in vivo* intervention experiments such as germ-free mouse colonization, fecal microbiota transplantation, and single-strain or metabolite reconstitution have bidirectionally validated causal associations between certain microbes/metabolites and fibrosis. Among these, the damaging effects of TMAO-mediated cardiac and skin fibrosis are supported by complete bidirectional causal evidence (Kim et al., 2022; Yang X. et al., 2025). The protective mechanisms of SCFAs, the AhR pathway, and the intestinal barrier have all demonstrated causal regulatory potential in various animal models, but human-level causal intervention evidence remains entirely absent (Nogal et al., 2021; Pan et al., 2025; Wang R. et al., 2024).

However, the conclusions of animal experiments are difficult to extrapolate directly to chronic fibrotic populations. Classical models such as chemical injury and bile duct ligation differ significantly from human fibrosis in disease duration, local inflammatory microenvironment, and host immunogenetic background; the same target intervention may produce opposite phenotypes in animals vs. humans (Sun et al., 2026; Qiu et al., 2024).

Current clinical observations are predominantly associative: Cross-sectional studies can yield only statistical correlations between microbiota/metabolites and fibrosis stage and are unable to distinguish whether dysbiosis induces fibrosis or whether organ pathology secondarily disrupts intestinal homeostasis (Loomba et al., 2017; Stec et al., 2023); longitudinal cohorts can provide temporal clues but still suffer from unadjusted confounders; and Mendelian randomization has been relatively underutilized in fibrosis research, with most reports confined to liver and intestinal diseases (Fan et al., 2024).

Throughout this review, we use the following standardized wording: Deterministic expressions such as “confirm” and “causal regulation” are used only for *in vivo* animal intervention experiments; the results from population cohorts and Mendelian randomization are uniformly described as “statistical associations” or “potential clues” to avoid over-interpretation of causality (Zhang et al., 2025; Fan et al., 2024). Future efforts should establish long-term prospective population cohorts combined with Mendelian randomization genetic analyses and standardized randomized controlled intervention trials to move the field from correlative observations to true causal validation of pathogenesis.

### 6.3 Exploration and challenges in microbiota-targeted therapeutic translation

The shared and organ-specific microbiota regulatory pathways summarized above provide a theoretical foundation for microbial intervention therapies, but translating basic animal experimental findings into clinical application faces multiple barriers (Henderson et al., 2020; Lavelle and Sokol, 2024). Existing intervention modalities fall into three categories: probiotics, fecal microbiota transplantation (FMT), and microbial metabolites/bacterial extracellular vesicles. Research in these areas has progressively moved from animal causal models to early clinical exploration (Ahn et al., 2025; Qiu et al., 2024).

#### 6.3.1 Probiotic interventions: sufficient causal evidence in animals but only associative evidence in humans

Germ-free colonization and single-strain reconstitution models have established complete bidirectional causal evidence chains: Bifidobacterium longum RAPO elevates butyrate to inhibit skin and pulmonary fibrosis (Park et al., 2024); Akkermansia muciniphila alleviates CCl4-induced liver fibrosis via the propionate–Keap1-Nrf2 pathway (Zhang et al., 2025; Oguri et al., 2024); supplementation with mixed butyrate-producing strains reduces myocardial collagen deposition in pressure-overload mice, whereas depletion of these taxa exacerbates fibrosis (Wang et al., 2022).

Human clinical trials can observe only correlations in noninvasive biomarkers, lacking pathological gold-standard validation: A 48-patient double-blind trial in metabolic dysfunction-associated steatotic liver disease (MASLD, formerly NAFLD) with a composite Lactobacillus + Bifidobacterium supplement showed only improved APRI scores, with no significant changes in hepatic collagen or steatosis (Abd El Hamid et al., 2024); a 350-patient MASLD cohort suggested that composite probiotics could lower serum fibrosis markers (Silva-Sperb et al., 2024); meta-analyses have confirmed that probiotics and prebiotics only mildly improve liver transaminases and steatosis indices (Chang et al., 2025; Kurban et al., 2025).

Intervention responses exhibit three layers of population stratification: (1) baseline microbiota: individuals with high abundance of butyrate-producing bacteria show better probiotic engraftment, whereas those with severe dysbiosis may require prior FMT or dietary intervention (Schnabl et al., 2025); (2) fibrosis stage: association signals are stable in early-stage (F0-F2) populations, whereas advanced (F3-F4) dense collagen barriers impede metabolite efficacy, with no histological improvement (Loomba et al., 2017); and (3) underlying disease: efficacy signals are most consistent in MASLD-associated liver fibrosis, whereas responses are generally poor in autoimmune biliary fibrosis, stricturing Crohn's intestinal fibrosis, and high-skin-score SSc fibrosis (Stec et al., 2023; Lavelle and Sokol, 2024; Nie et al., 2025).

Overall, composite strains containing Bifidobacterium show more stable effects, and single strains are insufficient to reverse advanced fibrosis; all the current trials rely on serum surrogate endpoints and cannot prove that microbiota interventions causally reverse collagen deposition in human organs (Chang et al., 2025; Abd El Hamid et al., 2024).

Negative clinical evidence should not be overlooked, as not all probiotic interventions yield observable benefits. One RCT in MASLD patients (NCT06074094) showed that 12-week Lactobacillus supplementation improved liver enzymes and BMI but had no significant effect on fibrosis scores (Abd El Hamid et al., 2024); another early-stage MASH study likewise failed to observe significant improvements in hepatic parameters with probiotics (Silva-Sperb et al., 2024). These negative results suggest that the regulatory effects of probiotics on fibrosis are influenced by strain selection, intervention duration, and baseline subject characteristics, and the applicable boundaries across different fibrotic stages and disease subtypes still require further definition.

#### 6.3.2 Fecal microbiota transplantation (FMT): complete bidirectional causal evidence in animals but only small-cohort associative clues in clinics

Various germ-free models have completed forward and reverse causal validation: mdr2^−/−^ germ-free mice with biliary fibrosis spontaneously develop severe bile duct fibrosis, and healthy microbiota transplantation significantly alleviates lesions (Awoniyi et al., 2023); bleomycin-induced pulmonary fibrosis mice receiving healthy donor FMT exhibit restored butyrate metabolism and suppressed epithelial–mesenchymal transition (Park et al., 2024; Zhou et al., 2025); and the transplantation of dysbiotic feces from uremic patients directly induces renal interstitial injury in wild-type mice (Wang Y. et al., 2024).

Currently, three registered fibrosis-related FMT trials have completed small-sample open-label cohorts or interim observations: NCT05821010 evaluates the improvement in the MASLD liver tissue SAF score with synbiotics combined with lyophilized fecal capsules, with full data not yet published (SYNCH Study Group, n.d.); NCT07477782 combines FMT with UDCA for PSC, with approximately one-third of open-label subjects showing ALP reduction, awaiting RCT confirmation (FARGO Study Group, n.d.); and NCT06024681 tracks colonization efficiency and hepatic fat changes in MASLD patients and is in the data analysis phase (FMT-NAFLD Study Group, n.d.).

Core translational bottlenecks: efficacy is highly dependent on the patient's baseline gut microbiota; donor screening and administration protocols lack standardization; existing studies have small sample sizes and short follow-up periods, lacking multicenter pathological-endpoint randomized controlled trials; and the evidence cannot yet confirm that FMT can reverse fibrosis in humans (Schnabl et al., 2025; Craven et al., 2020).

The results of negative FMT trials also provide valuable reference data. A 2025 double-blind RCT revealed that three consecutive FMT administrations failed to significantly improve hepatic steatosis (MRI-PDFF, *p* = 0.50) or glucose tolerance in MASLD patients, and no stable donor engraftment or microbial signature predictive of clinical response was identified (Craven et al., 2020). Several other MASLD-related FMT RCTs have also reported no significant improvements in liver histology or metabolic parameters (Craven et al., 2020). In PSC populations, FMT only modestly increased gut microbial diversity without substantial changes in fecal bile acid profiles (Little et al., 2020); probiotic/synbiotic supplementation in cystic fibrosis patients also failed to improve lung function or reduce the frequency of acute pulmonary exacerbations (Coffey et al., 2020). These negative results suggest that FMT efficacy is highly modulated by donor–recipient microbiota compatibility, baseline microbial diversity, and intervention frequency, and the conditions suitable for different fibrotic populations still require systematic exploration. Together, positive and negative studies constitute a complete evidence landscape: the efficacy of microbiota interventions exhibits strong individual heterogeneity, and future efforts require rigorous patient stratification and unified standardized protocols to precisely delineate the beneficiary populations and applicable boundaries of interventions.

#### 6.3.3 Overall translational summary

Four categories of animal experiments—germ-free colonization, strain reconstitution, FMT, and metabolite administration—have confirmed that the microbiota can causally regulate fibrosis (Zhang et al., 2025; Wang et al., 2022; Park et al., 2024; Zhou et al., 2025); however, all clinical studies to date have observed only correlations in biochemical and imaging indicators, without evidence of organ pathological intervention, and cannot establish therapeutic causality at the human level (Loomba et al., 2017; Stec et al., 2023; Abd El Hamid et al., 2024). Emerging mediators such as bacterial extracellular vesicles, sphingolipid metabolism, and metabolic reprogramming (which are detailed mechanistically in Section 3.5) remain at an even earlier translational stage, with no human interventional data and, for most fibrosis types, limited human correlational evidence (Ahn et al., 2025; Wang Y. et al., 2024; Li Y. et al., 2026).

Microbiota interventions have a clear stage-dependent efficacy boundary: In early fibrosis, characterized by inflammation and fibroblast activation, microbial metabolites can exert preventive effects; in advanced stages, where collagen is extensively cross-linked into a dense extracellular matrix, metabolites cannot degrade mature collagen, and therapeutic efficacy is minimal (Rockey et al., 2015; Lavelle and Sokol, 2024). Most animal studies adopt preventive dosing during the fibrosis induction phase, yielding more pronounced intervention effects, whereas postfibrosis administration shows relatively limited efficacy (Zhang et al., 2025; Sun et al., 2026). Therefore, microbiota-targeted therapies are positioned as early prevention strategies in high-risk patients and as adjunctive combination strategies rather than as standalone replacement therapy for advanced fibrosis (Chang et al., 2025; Nie et al., 2025). Future clinical trials should stratify enrollment by fibrosis stage, distinguish slowing progression and reversing fibrosis as two independent endpoints, and simultaneously develop multistrain and metabolite combination intervention strategies (Abd El Hamid et al., 2024; Craven et al., 2020).

## 7 Conclusions and perspectives

The regulation of multiorgan fibrosis by the gut microbiota follows a dual-layer framework of shared regulation and organ-specific differentiation (Henderson et al., 2020; Lin et al., 2025). The four core metabolic–immune pathways—short-chain fatty acid signaling, the tryptophan–AhR axis, intestinal barrier integrity, and bile acid/FXR signaling—constitute the common underlying mechanisms of microbiota-mediated susceptibility to systemic organ fibrosis (Nogal et al., 2021; Lavelle and Sokol, 2024; Roager and Licht, 2018; Pan et al., 2025). Antifibrotic commensals (Akkermansia, Lactobacillus) and profibrotic pathogens (Enterobacteriaceae, etc.) bidirectionally regulate distal organ collagen homeostasis via multiple gut–organ communication axes (Zhang et al., 2025; Oguri et al., 2024; Ahn et al., 2025).

Organ-specific regulation is shaped jointly by anatomical structures, resident cell types, and local microenvironments, with each organ forming its own non-interchangeable dominant pathway: The hepatobiliary system is regulated primarily by bile acid/FXR signaling (Shi et al., 2023; Xiao et al., 2025; Kasahara et al., 2023); intestinal fibrosis relies on SCFAs combined with intestinal barrier protection as its defensive mechanism (Nogal et al., 2021; Saha et al., 2025; Li X. et al., 2024); myocardial fibrosis possesses the unique profibrotic TMAO–JAK2–STAT3 pathway (Yang X. et al., 2025; Zhu et al., 2016); pulmonary fibrosis involves multiple concurrent pathways with PEA as an organ-specific protective mediator (Zhou et al., 2025; Yang J. et al., 2025); and skin fibrosis superimposes circulating microbial signals with local Piezo1 mechanosensory pathways to form a dual regulatory network (Kim et al., 2022; Liao et al., 2025).

At the cross-organ level, a unified pathological cascade exists: Gut dysbiosis sequentially drives local chronic inflammation, pathological fibroblast activation, and excessive extracellular matrix deposition (Rockey et al., 2015; Saha et al., 2025; Sun et al., 2026). Intervention models, including germ-free colonization, single-strain/metabolite reconstitution, and FMT, have fully confirmed the value of the microbiota and its derivatives as therapeutic targets for fibrosis (Zhang et al., 2025; Wang et al., 2022; Park et al., 2024; Awoniyi et al., 2023). However, current population cohorts can reveal only statistical associations between microbial biomarkers and fibrosis stage (Loomba et al., 2017; Stec et al., 2023); prospective clinical trials with organ histopathology as the primary endpoint are lacking, and it remains unproven that microbiota interventions can reverse established fibrotic lesions in humans (Coffey et al., 2020; Tegegne and Savidge, 2025).

At present, the clinical translation of microbiota-targeted fibrosis therapies faces multiple inherent bottlenecks: Substantial heterogeneity exists between the disease courses and immunoinflammatory backgrounds in animal models and in human chronic fibrotic diseases (Sun et al., 2026; Qiu et al., 2024); most clinical observations are single-center cross-sectional studies with small sample sizes, making it difficult to distinguish causality from confounding (Loomba et al., 2017; Stec et al., 2023); intervention responses are highly dependent on the recipient's baseline gut microbiota (Craven et al., 2020; Tegegne and Savidge, 2025); and single-strain interventions have limited efficacy (Chang et al., 2025; Coffey et al., 2020). The development potential of pathways is clearly stratified: SCFAs, AhR, and bile acids/FXR exhibit broad-spectrum antifibrotic activity in multiple animal models (Nogal et al., 2021; Pan et al., 2025; Wang R. et al., 2024), whereas organ-specific targets such as PEA, TMAO, and Piezo1 are better suited for stratified precision interventions (Kim et al., 2022; Zhou et al., 2025; Liao et al., 2025). However, human interventional evidence remains severely lacking (Fan et al., 2024; Coffey et al., 2020).

For future basic and clinical research, the field should advance along two core directions: causal confirmation and translational application. (1) Conduct targeted intervention trials for core pathways in each organ, advancing stratified validation of small-molecule metabolic modulators such as FXR agonists and TMAO synthesis inhibitors (Xiao et al., 2025; Zhu et al., 2016; Craven et al., 2020). (2) Integrate metagenomic and metabolomic multiomics with Mendelian randomization analysis to transform cross-sectional correlational findings into actionable causal hypotheses (Fan et al., 2024; Tegegne and Savidge, 2025). (3) Conduct standardized, multicenter randomized controlled trials with histopathology or quantitative imaging as hard endpoints to complete human validation, driving microbiota-related mechanisms from basic causal animal research into precision-stratified prevention and treatment strategies for multiorgan fibrosis (Loomba et al., 2017; Coffey et al., 2020; Tegegne and Savidge, 2025).

## Glossary

APRI, AST-to-platelet ratio index; AhR, aryl hydrocarbon receptor; ARNT, aryl hydrocarbon receptor nuclear translocator; BEV, bacterial extracellular vesicle; CMR ECV, cardiac magnetic resonance extracellular volume; COL1A1, collagen type I alpha 1 chain; CYP1A1, cytochrome P450 1A1; CYP7A1, cholesterol 7α-hydroxylase; DRE, dioxin response element; ECM, extracellular matrix; ERK, extracellular signal-regulated kinase; FGF15, fibroblast growth factor 15; FIB-4, fibrosis-4 index; FMO, flavin-containing monooxygenase; FMT, fecal microbiota transplantation; FXR, farnesoid X receptor; GPCR, G protein-coupled receptor; GPR41/43, G protein-coupled receptor 41/43; GSDMD, gasdermin D; HDAC, histone deacetylase; HIF-1α, hypoxia-inducible factor 1α; HPA, hypothalamic–pituitary–adrenal axis; HSC, hepatic stellate cell; IBD, inflammatory bowel disease; IL, interleukin; ILD, interstitial lung disease; IPF, idiopathic pulmonary fibrosis; JAK2, Janus kinase 2; Keap1, kelch-like ECH-associated protein 1; KL-6, Krebs von den Lungen-6; LPS, lipopolysaccharide; M2b, M2b macrophage; MAPK, mitogen-activated protein kinase; MASH, metabolic dysfunction-associated steatohepatitis; MASLD, metabolic dysfunction-associated steatotic liver disease; MDSC, myeloid-derived suppressor cell; MMP, matrix metalloproteinase; MR, Mendelian randomization; mTOR, mechanistic target of rapamycin; NF-κB, nuclear factor kappa B; NLRP3, NOD-, LRR- and pyrin domain-containing protein 3; Nrf2, nuclear factor erythroid 2-related factor 2; PBC, primary biliary cholangitis; PDC-E2, pyruvate dehydrogenase complex E2 subunit; PEA, palmitoylethanolamide; PERK, protein kinase RNA-like endoplasmic reticulum kinase; Piezo1, piezo-type mechanosensitive ion channel component 1; PPAR, peroxisome proliferator-activated receptor; PPARD, peroxisome proliferator-activated receptor delta; PSC, primary sclerosing cholangitis; RCT, randomized controlled trial; SAF score, Steatosis, Activity, and Fibrosis score; SCFA, short-chain fatty acid; SHP, small heterodimer partner; SMPD3, sphingomyelin phosphodiesterase 3; SP-D, surfactant protein D; STAT3, signal transducer and activator of transcription 3; TCDD, 2,3,7,8-tetrachlorodibenzo-p-dioxin; TDO2, tryptophan 2,3-dioxygenase; TGF-β, transforming growth factor beta; TGFBR1, transforming growth factor beta receptor 1; TGR5, Takeda G protein-coupled receptor 5; TLR, toll-like receptor; TLR4, toll-like receptor 4; TMAO, trimethylamine N-oxide; Treg, regulatory T cell; UDCA, ursodeoxycholic acid; YAP, Yes-associated protein; YBX1, Y-box binding protein 1.
